# Supplementary material for: Redundancy of macrobenthic functional traits boosts resilience to a simulated heatwave
Source: PLoS One. 2026 Jan 12;21(1):e0340819. doi: 10.1371/journal.pone.0340819 (PMC12795362; doi:10.1371/journal.pone.0340819)
Supplement: S1 Table — Fuzzy coding was applied to each of the taxa ranging from 0 to 1. (DOCX) [file pone.0340819.s001.docx]

**S1 Table.** Macrobenthic taxa and functional trait modalities assessed for the simulated heatwave in situ experiment. Fuzzy coding was applied to each of the taxa ranging from 0 to 1.

| Taxa | Biodiffusor | Bioirrigator | No bioturbation | Surface modifier | Large (>20mm) | Medium (5-20mm) | Small (0.5-5mm) | Deposit feeder | Filter/suspension | Grazer/scraper | Omnivore | Predator | Scavenger/opportunist | Sub-surface deposit feeder | Attached | Burrower | Free living / Surface crawler | Parasite / Commensal | Tube dwelling | Irregular | Round / Globulose | Streamlined | Vermiform | Burrower | Crawler | None | Swimmer | Attached | Bentho-pelagic | Epibenthic | Crevices, stones, shells | Deeper than 3cm | Surface shallow <3cm |
| --- | --- | --- | --- | --- | --- | --- | --- | --- | --- | --- | --- | --- | --- | --- | --- | --- | --- | --- | --- | --- | --- | --- | --- | --- | --- | --- | --- | --- | --- | --- | --- | --- | --- |
| *Arthritica sp.* | 0 | 0 | 0 | 1 | 0 | 0 | 1 | 0.5 | 0.5 | 0 | 0 | 0 | 0 | 0 | 0 | 1 | 0 | 0 | 0 | 0 | 1 | 0 | 0 | 1 | 0 | 0 | 0 | 0 | 0 | 0 | 0 | 0 | 1 |
| *Austrovenus stutchburyi* | 0 | 0.5 | 0 | 0.5 | 1 | 0 | 0 | 0 | 1 | 0 | 0 | 0 | 0 | 0 | 0 | 1 | 0 | 0 | 0 | 0 | 1 | 0 | 0 | 1 | 0 | 0 | 0 | 0 | 0 | 0 | 0 | 0 | 1 |
| *Capitella sp.* | 1 | 0 | 0 | 0 | 0 | 0 | 1 | 0.5 | 0 | 0 | 0 | 0 | 0 | 0.5 | 0 | 0.5 | 0 | 0 | 1 | 0 | 0 | 0 | 1 | 1 | 0 | 0 | 0 | 0 | 0 | 0 | 0 | 1 | 0 |
| *Ceratonereis sp.* | 0.33 | 0.3 | 0 | 0.3 | 0 | 1 | 0 | 0.25 | 0 | 0.25 | 0 | 0.3 | 0.25 | 0 | 0 | 1 | 0 | 0 | 0 | 0 | 0 | 0 | 1 | 0 | 1 | 0 | 0 | 0 | 0 | 0 | 0 | 1 | 1 |
| Chaetognatha | 0 | 0 | 1 | 0 | 0 | 0 | 1 | 0 | 0 | 0 | 0 | 1 | 0 | 0 | 0 | 0 | 1 | 0 | 0 | 0 | 0 | 0 | 1 | 0 | 0 | 0 | 1 | 1 | 1 | 0 | 0 | 0 | 0 |
| Chironomidae larvae | 0 | 0 | 0 | 1 | 0 | 0 | 1 | 0 | 0 | 0 | 0 | 0 | 1 | 0 | 0 | 0 | 1 | 0 | 0 | 0 | 0 | 0 | 1 | 0 | 1 | 0 | 0 | 0 | 0 | 1 | 0 | 0 | 0 |
| *Colurostylis lemurum* | 0 | 0 | 0 | 1 | 0 | 0 | 1 | 1 | 0 | 0 | 0 | 0 | 0 | 0 | 0 | 1 | 0 | 0 | 0 | 0 | 0 | 1 | 0 | 1 | 0 | 0 | 0 | 0 | 0 | 0 | 0 | 0 | 1 |
| *Cominella glandiformis* | 0 | 0 | 0 | 1 | 1 | 0 | 0 | 0 | 0 | 0 | 0 | 0.5 | 0.5 | 0 | 0 | 0 | 1 | 0 | 0 | 0 | 1 | 0 | 0 | 0 | 1 | 0 | 0 | 0 | 0 | 0 | 0 | 0 | 1 |
| *Copepoda* | 0 | 0 | 1 | 0 | 0 | 0 | 1 | 0 | 0 | 0 | 0 | 1 | 0 | 0 | 0 | 0 | 1 | 0 | 0 | 0 | 0 | 1 | 0 | 0 | 0 | 0 | 1 | 0 | 1 | 0 | 0 | 0 | 0 |
| *Exosphaeroma planulum* | 0 | 0 | 0 | 1 | 0 | 0 | 1 | 0 | 0 | 0 | 0 | 0.5 | 0.5 | 0 | 0 | 0 | 0.8 | 0 | 0 | 0 | 0 | 1 | 0 | 0 | 1 | 0 | 0 | 0 | 0 | 0 | 0 | 0 | 1 |
| *Halicarcinus whitei* | 0 | 0 | 0 | 1 | 0 | 1 | 0 | 0 | 0 | 0 | 0 | 0.5 | 0.5 | 0 | 0 | 0.3 | 0.8 | 0 | 0 | 1 | 0.5 | 0 | 0 | 0 | 1 | 0 | 0 | 0 | 0 | 1 | 0 | 0 | 0 |
| *Halopyrgus pupoides* | 0 | 0 | 0 | 1 | 0 | 0 | 1 | 0 | 0 | 0 | 0 | 0.5 | 0.5 | 0 | 0 | 0 | 1 | 0 | 0 | 0 | 1 | 0 | 0 | 0 | 1 | 0 | 0 | 0 | 0 | 1 | 0 | 0 | 0 |
| *Hemiplax hirtipes* | 0.5 | 0 | 0 | 0.5 | 1 | 0 | 0 | 0.33 | 0 | 0 | 0 | 0.3 | 0.33 | 0 | 0 | 0.5 | 0.5 | 0 | 0 | 1 | 0.5 | 0 | 0 | 0 | 1 | 0 | 0 | 0 | 0 | 0 | 0 | 1 | 1 |
| *Josephosella awa* | 0 | 0 | 0 | 1 | 0 | 0 | 1 | 0.5 | 0 | 0.25 | 0 | 0 | 0.25 | 0 | 0 | 0 | 1 | 0 | 0 | 0 | 0 | 1 | 0 | 0 | 1 | 0 | 0 | 0 | 0 | 1 | 0 | 0 | 0 |
| *Microphthalmus riseri* | 0 | 0 | 0 | 1 | 0 | 0 | 1 | 0 | 0 | 0 | 0 | 0.5 | 0.5 | 0 | 0 | 0 | 0.8 | 0 | 0 | 0 | 0 | 0 | 1 | 0 | 1 | 0 | 0.3 | 0 | 0 | 1 | 0 | 0 | 0 |
| *Microspio maori* | 0.5 | 0 | 0 | 0.5 | 0 | 0 | 1 | 1 | 0 | 0 | 0 | 0 | 0 | 0 | 0 | 0 | 0 | 0 | 1 | 0 | 0 | 0 | 1 | 1 | 0 | 0 | 0 | 0 | 0 | 0 | 0 | 1 | 1 |
| Nemertea | 0.5 | 0 | 0 | 0.5 | 0 | 0 | 1 | 0 | 0 | 0 | 0 | 0.5 | 0.5 | 0 | 0 | 0 | 1 | 0 | 0 | 0 | 0 | 0 | 1 | 0 | 1 | 0 | 0 | 0 | 0 | 0 | 0 | 1 | 1 |
| *Nicon aestuariensis* | 0.5 | 0 | 0 | 0.5 | 0 | 1 | 0 | 0.25 | 0 | 0.25 | 0 | 0.3 | 0.25 | 0 | 0 | 0 | 1 | 0 | 0 | 0 | 0 | 0 | 1 | 0 | 1 | 0 | 0 | 0 | 0 | 0 | 0 | 1 | 1 |
| Oligochaeta | 0.33 | 0.3 | 0 | 0.3 | 0 | 0 | 1 | 0.33 | 0 | 0 | 0 | 0.3 | 0.33 | 0 | 0 | 0.5 | 0.5 | 0 | 0 | 0 | 0 | 0 | 1 | 1 | 0 | 0 | 0 | 0 | 0 | 0 | 0 | 1 | 1 |
| *Paracalliope novizealandiae* | 0 | 0 | 0 | 1 | 0 | 0 | 1 | 0.5 | 0.25 | 0.25 | 0 | 0 | 0 | 0 | 0 | 0 | 1 | 0 | 0 | 0 | 0 | 1 | 0 | 0 | 1 | 0 | 0 | 0 | 0 | 1 | 0 | 0 | 0 |
| *Paracorophium excavatum* | 0.5 | 0 | 0 | 0.5 | 0 | 1 | 0 | 0 | 1 | 0 | 0 | 0 | 0 | 0 | 0 | 0 | 0 | 0 | 1 | 0 | 0 | 1 | 0 | 1 | 1 | 0 | 0 | 0 | 0 | 1 | 0 | 0 | 1 |
| *Paradoneis lyra* | 0.33 | 0.3 | 0 | 0.3 | 0 | 0 | 1 | 0.5 | 0 | 0.5 | 0 | 0 | 0 | 0 | 0 | 1 | 0 | 0 | 0 | 0 | 0 | 0 | 1 | 1 | 0 | 0 | 0 | 0 | 0 | 0 | 0 | 0 | 0 |
| *Perinereis vallata* | 0.33 | 0.3 | 0 | 0.3 | 0 | 1 | 0 | 0 | 0 | 0 | 0 | 0.5 | 0.5 | 0 | 0 | 0 | 1 | 0 | 0 | 0 | 0 | 0 | 1 | 0 | 1 | 0 | 0 | 0 | 0 | 0 | 0 | 1 | 1 |
| *Potamopyrgus estuarinus* | 0 | 0 | 0 | 1 | 0 | 0 | 1 | 0 | 0 | 0 | 0 | 0.5 | 0.5 | 0 | 0 | 0 | 1 | 0 | 0 | 0 | 1 | 0 | 0 | 0 | 1 | 0 | 0 | 0 | 0 | 1 | 0 | 0 | 0 |
| *Scolecolepides benhami* | 1 | 0 | 0 | 0 | 0 | 1 | 0 | 1 | 0 | 0 | 0 | 0 | 0 | 0 | 0 | 0 | 0 | 0 | 1 | 0 | 0 | 0 | 1 | 1 | 0 | 0 | 0 | 0 | 0 | 0 | 0 | 1 | 1 |
