# Supplementary material for: Redundancy of macrobenthic functional traits boosts resilience to a simulated heatwave
Source: PLoS One. 2026 Jan 12;21(1):e0340819. doi: 10.1371/journal.pone.0340819 (PMC12795362; doi:10.1371/journal.pone.0340819)
Supplement: S2 Table — (DOCX) [file pone.0340819.s002.docx]

**S2 Table.** Macrobenthic taxa list showing the taxa recorded in the simulated heatwave in-situ experiment.

| **Phylum** | **Class** | **Order/Subclass** | **Family** | **TAXA** |
| --- | --- | --- | --- | --- |
| Arthropoda | Malacostraca | Amphipoda | Melitidae | *Melita awa* |
| Arthropoda | Malacostraca | Amphipoda | Paracalliopidae | *Paracalliope novizealandiae* |
| Arthropoda | Malacostraca | Amphipoda | Corophiidae | *Paracorophium excavatum* |
| Arthropoda | Malacostraca | Cumacea | Diastylidae | *Colurostylis lemurum* |
| Arthropoda | Malacostraca | Decapoda | Hymenosomatidae | *Halicarcinus whitei* |
| Arthropoda | Malacostraca | Decapoda | Macrophthalmidae | *Hemiplax hirtipes* |
| Arthropoda | Insecta | Diptera | Chironomidae | Chironomidae |
| Arthropoda | Malacostraca | Isopoda | Sphaeromatidae | *Exosphaeroma planulum* |
| Mollusca | Gastropoda | Littorinimorpha | Tateidae | *Halopyrgus pupoides* |
| Mollusca | Gastropoda | Littorinimorpha | Tateidae | *Potamopyrgus estuarinus* |
| Mollusca | Gastropoda | Neogastropoda | Buccinulidae | *Cominella glandiformis* |
| Annelida | Clitellata | Oligochaeta |  | Oligochaeta |
| Annelida | Polychaeta | Phyllodocida | Nereididae | *Ceratonereis sp.* |
| Annelida | Polychaeta | Phyllodocida | Microphthalmidae | *Microphthalmus riseri* |
| Annelida | Polychaeta | Phyllodocida | Nereididae | *Nicon aestuariensis* |
| Annelida | Polychaeta | Phyllodocida | Nereididae | *Perinereis vallata* |
| Annelida | Polychaeta | Scolecida | Capitellidae | *Capitella spp.* |
| Annelida | Polychaeta | Scolecida | Paraonidae | *Paradoneis lyra* |
| Annelida | Polychaeta | Spionida | Spionidae | *Microspio maori* |
| Annelida | Polychaeta | Spionida | Spionidae | *Scolecolepides benhami* |
| Mollusca | Bivalvia | Venerida | Lasaeidae | *Arthritica sp.* |
| Mollusca | Bivalvia | Venerida | Veneridae | *Austrovenus stutchburyi* |
| Arthropoda | Copepoda |  |  | Copepoda |
| Chaetognatha |  |  |  | Chaetognatha |
| Nemertea |  |  |  | Nemertea |
