# Supplementary material for: Redundancy of macrobenthic functional traits boosts resilience to a simulated heatwave
Source: PLoS One. 2026 Jan 12;21(1):e0340819. doi: 10.1371/journal.pone.0340819 (PMC12795362; doi:10.1371/journal.pone.0340819)
Supplement: S3 Table — (DOCX) [file pone.0340819.s003.docx]

**S3 Table.** Macrobenthic abundance (organisms per core^-1^) collected in the control (C), short (S), and long (L) duration treatments from the simulated heatwave in situ experiment.

| Taxa | *Arthritica sp.* | *Austrovenus stutchburyi* | *Capitella sp.* | *Ceratonereis sp.* | Chaetognatha | Chironomidae larvae | *Colurostylis lemurum* | *Cominella glandiformis* | Copepoda | *Exosphaeroma planulum* | *Halicarcinus whitei* | *Halopyrgus pupoides* | *Hemiplax hirtipes* | *Josephosella awa* | *Microphthalmus riseri* | *Microspio maori* | Nemertea | *Nicon aestuariensis* | Oligochaeta | *Paracalliope novizealandiae* | *Paracorophium excavatum* | *Paradoneis lyra* | *Perinereis vallata* | *Potamopyrgus estuarinus* | *Scolecolepides benhami* |
| --- | --- | --- | --- | --- | --- | --- | --- | --- | --- | --- | --- | --- | --- | --- | --- | --- | --- | --- | --- | --- | --- | --- | --- | --- | --- |
| C.1 | 13 | 0 | 6 | 15 | 0 | 0 | 0 | 0 | 0 | 0 | 0 | 7 | 1 | 0 | 0 | 3 | 1 | 1 | 23 | 1 | 290 | 2 | 0 | 2 | 3 |
| C.2 | 41 | 0 | 4 | 17 | 0 | 0 | 0 | 0 | 0 | 1 | 0 | 3 | 1 | 2 | 0 | 17 | 0 | 2 | 26 | 0 | 432 | 0 | 0 | 7 | 2 |
| C.3 | 7 | 0 | 5 | 12 | 0 | 0 | 0 | 0 | 0 | 1 | 1 | 7 | 1 | 1 | 0 | 8 | 0 | 0 | 18 | 0 | 367 | 0 | 0 | 0 | 6 |
| C.4 | 0 | 0 | 8 | 13 | 0 | 0 | 0 | 0 | 14 | 0 | 0 | 0 | 1 | 9 | 0 | 12 | 0 | 4 | 12 | 0 | 328 | 0 | 0 | 0 | 4 |
| C.5 | 36 | 0 | 0 | 5 | 0 | 0 | 0 | 0 | 0 | 0 | 0 | 0 | 5 | 15 | 0 | 2 | 0 | 3 | 1 | 0 | 352 | 0 | 0 | 0 | 3 |
| C.6 | 48 | 0 | 1 | 14 | 0 | 0 | 0 | 1 | 0 | 0 | 0 | 0 | 1 | 25 | 0 | 2 | 0 | 6 | 5 | 0 | 207 | 0 | 0 | 0 | 5 |
| C.7 | 41 | 0 | 0 | 9 | 0 | 0 | 0 | 0 | 0 | 0 | 0 | 1 | 3 | 9 | 0 | 0 | 0 | 4 | 3 | 0 | 219 | 0 | 0 | 0 | 7 |
| C.8 | 9 | 0 | 1 | 6 | 0 | 0 | 0 | 0 | 0 | 0 | 1 | 1 | 1 | 1 | 0 | 7 | 0 | 2 | 1 | 0 | 235 | 0 | 0 | 0 | 4 |
| C.9 | 12 | 0 | 4 | 12 | 0 | 0 | 0 | 0 | 0 | 0 | 0 | 2 | 0 | 0 | 0 | 14 | 0 | 7 | 11 | 0 | 252 | 0 | 0 | 0 | 3 |
| C.10 | 15 | 0 | 3 | 18 | 0 | 1 | 0 | 0 | 0 | 1 | 0 | 4 | 0 | 6 | 0 | 4 | 0 | 5 | 5 | 2 | 259 | 0 | 0 | 2 | 8 |
| S.1 | 26 | 0 | 3 | 9 | 0 | 0 | 0 | 0 | 0 | 1 | 0 | 6 | 2 | 2 | 1 | 10 | 1 | 1 | 39 | 0 | 196 | 0 | 0 | 7 | 5 |
| S.2 | 5 | 0 | 0 | 6 | 1 | 0 | 0 | 0 | 2 | 0 | 0 | 7 | 2 | 0 | 0 | 3 | 0 | 3 | 6 | 0 | 205 | 0 | 0 | 0 | 4 |
| S.3 | 21 | 0 | 2 | 23 | 0 | 0 | 0 | 0 | 0 | 0 | 0 | 3 | 0 | 0 | 0 | 7 | 0 | 2 | 21 | 0 | 267 | 0 | 0 | 1 | 6 |
| S.4 | 23 | 0 | 6 | 8 | 0 | 0 | 0 | 0 | 0 | 0 | 0 | 14 | 3 | 3 | 0 | 5 | 0 | 4 | 6 | 0 | 231 | 0 | 0 | 3 | 2 |
| S.5 | 5 | 0 | 0 | 9 | 0 | 0 | 0 | 1 | 0 | 0 | 0 | 2 | 1 | 3 | 0 | 8 | 0 | 2 | 2 | 0 | 169 | 0 | 0 | 0 | 3 |
| S.6 | 30 | 0 | 6 | 12 | 0 | 0 | 0 | 0 | 0 | 0 | 0 | 10 | 0 | 8 | 1 | 6 | 1 | 3 | 29 | 0 | 92 | 0 | 0 | 3 | 4 |
| S.7 | 19 | 1 | 1 | 8 | 0 | 0 | 0 | 0 | 0 | 0 | 0 | 2 | 1 | 0 | 0 | 12 | 0 | 10 | 5 | 0 | 150 | 0 | 0 | 2 | 1 |
| S.8 | 20 | 0 | 6 | 11 | 0 | 0 | 0 | 0 | 0 | 0 | 0 | 7 | 0 | 1 | 0 | 6 | 0 | 6 | 13 | 0 | 119 | 0 | 0 | 0 | 5 |
| S.9 | 24 | 0 | 15 | 0 | 0 | 0 | 0 | 0 | 0 | 0 | 0 | 4 | 1 | 2 | 1 | 1 | 0 | 18 | 10 | 0 | 157 | 0 | 1 | 0 | 4 |
| S.10 | 6 | 0 | 0 | 9 | 0 | 0 | 1 | 0 | 1 | 0 | 0 | 1 | 4 | 2 | 0 | 4 | 0 | 7 | 2 | 0 | 145 | 0 | 0 | 0 | 4 |
| L.1 | 23 | 0 | 2 | 13 | 0 | 0 | 0 | 0 | 1 | 0 | 0 | 3 | 2 | 6 | 0 | 13 | 0 | 1 | 35 | 0 | 82 | 0 | 0 | 3 | 3 |
| L.2 | 21 | 0 | 3 | 16 | 0 | 0 | 0 | 0 | 0 | 0 | 0 | 14 | 1 | 3 | 0 | 8 | 0 | 3 | 14 | 0 | 317 | 0 | 0 | 4 | 4 |
| L.3 | 7 | 0 | 3 | 24 | 0 | 0 | 0 | 0 | 0 | 1 | 0 | 22 | 3 | 2 | 0 | 6 | 0 | 4 | 11 | 0 | 198 | 0 | 0 | 2 | 1 |
| L.4 | 34 | 0 | 1 | 15 | 0 | 0 | 0 | 0 | 0 | 1 | 0 | 7 | 3 | 12 | 0 | 4 | 0 | 3 | 12 | 0 | 250 | 0 | 0 | 3 | 4 |
| L.5 | 7 | 0 | 6 | 21 | 0 | 0 | 0 | 0 | 2 | 0 | 0 | 7 | 2 | 3 | 0 | 12 | 1 | 3 | 17 | 0 | 201 | 0 | 0 | 0 | 2 |
| L.6 | 39 | 0 | 5 | 18 | 0 | 0 | 0 | 0 | 0 | 1 | 0 | 6 | 1 | 13 | 0 | 6 | 2 | 4 | 12 | 0 | 183 | 0 | 0 | 2 | 4 |
| L.7 | 9 | 0 | 3 | 15 | 0 | 0 | 0 | 1 | 0 | 1 | 0 | 11 | 2 | 0 | 0 | 5 | 0 | 3 | 7 | 0 | 123 | 0 | 0 | 3 | 8 |
| L.8 | 19 | 0 | 13 | 9 | 0 | 0 | 0 | 1 | 0 | 0 | 1 | 6 | 1 | 0 | 0 | 6 | 0 | 5 | 3 | 0 | 136 | 0 | 0 | 1 | 7 |
| L.9 | 33 | 0 | 1 | 8 | 0 | 0 | 0 | 0 | 0 | 0 | 0 | 1 | 0 | 4 | 0 | 3 | 0 | 7 | 5 | 0 | 137 | 0 | 0 | 1 | 6 |
| L.10 | 19 | 0 | 1 | 16 | 0 | 0 | 0 | 0 | 0 | 0 | 0 | 10 | 2 | 1 | 0 | 13 | 0 | 4 | 23 | 0 | 84 | 0 | 0 | 1 | 14 |
