# Supplementary material for: Redundancy of macrobenthic functional traits boosts resilience to a simulated heatwave
Source: PLoS One. 2026 Jan 12;21(1):e0340819. doi: 10.1371/journal.pone.0340819 (PMC12795362; doi:10.1371/journal.pone.0340819)
Supplement: S4 Table — (DOCX) [file pone.0340819.s004.docx]

**S4 Table.** Summary statistics of the macrobenthic taxa metrics recorded in simulated heatwave in situ experiment.

|  | **Richness** | | | **Abundance** (organisms per core^-1^) | | | **Diversity (H’)** | | |
| --- | --- | --- | --- | --- | --- | --- | --- | --- | --- |
| **Treatment** | **Mean** | **Max** | **Min** | **Mean** | **Max** | **Min** | **Mean** | **Max** | **Min** |
| **Control** | 15.65 | 23.00 | 9.00 | 368.45 | 895.00 | 188.00 | 1.51 | 2.39 | 0.64 |
| **Long** | 16.20 | 26.00 | 11.00 | 301.10 | 430.00 | 187.00 | 1.75 | 2.31 | 1.00 |
| **Short** | 14.55 | 23.00 | 10.00 | 258.25 | 702.00 | 105.00 | 1.59 | 2.35 | 0.79 |
