# Supplementary material for: Redundancy of macrobenthic functional traits boosts resilience to a simulated heatwave
Source: PLoS One. 2026 Jan 12;21(1):e0340819. doi: 10.1371/journal.pone.0340819 (PMC12795362; doi:10.1371/journal.pone.0340819)
Supplement: S5 Table — (DOCX) [file pone.0340819.s005.docx]

**S5 Table.** Summary table of the macrobenthic CWM (Community-level Weighted Means) of trait values recorded in simulated heatwave in situ experiment.

| **Treatment** | Biodiffusor | Bioirrigator | No bioturbation | Surface modifier | Large (>20mm) | Medium (5-20mm) | Small (0.5-5mm) | Deposit feeder | Filter/suspension | Grazer/scraper | Omnivore | Predator | Scavenger/opportunist | Sub-surface deposit feeder | Attached | Burrower | Free living / Surface crawler | Parasite / Commensal | Tube dwelling | Irregular | Round / Globulose | Streamlined | Vermiform | Burrower | Crawler | None | Swimmer |
| --- | --- | --- | --- | --- | --- | --- | --- | --- | --- | --- | --- | --- | --- | --- | --- | --- | --- | --- | --- | --- | --- | --- | --- | --- | --- | --- | --- |
| Control | 0.452 | 0.020 | 0.000 | 0.524 | 0.000 | 1.000 | 0.000 | 0.097 | 0.821 | 0.016 | 0.0 | 0.029 | 0.031 | 0.004 | 0.115 | 0.060 | 0.000 | 0.825 | 0.523 | 0.474 | 0.000 | 0.003 | 0.000 | 0.003 | 0.425 | 0.061 | 0.510 |
| Short | 0.442 | 0.031 | 0.000 | 0.525 | 0.000 | 1.000 | 0.000 | 0.128 | 0.739 | 0.018 | 0.0 | 0.053 | 0.053 | 0.008 | 0.151 | 0.097 | 0.000 | 0.752 | 0.536 | 0.462 | 0.000 | 0.002 | 0.000 | 0.002 | 0.390 | 0.099 | 0.509 |
| Long | 0.426 | 0.040 | 0.000 | 0.532 | 0.000 | 1.000 | 0.000 | 0.156 | 0.679 | 0.023 | 0.0 | 0.065 | 0.067 | 0.007 | 0.188 | 0.111 | 0.000 | 0.701 | 0.535 | 0.464 | 0.000 | 0.001 | 0.000 | 0.001 | 0.376 | 0.116 | 0.506 |
