# Supplementary material for: Redundancy of macrobenthic functional traits boosts resilience to a simulated heatwave
Source: PLoS One. 2026 Jan 12;21(1):e0340819. doi: 10.1371/journal.pone.0340819 (PMC12795362; doi:10.1371/journal.pone.0340819)
Supplement: S6 Table — (DOCX) [file pone.0340819.s006.docx]

**S6 Table.** Summary statistics of the macrobenthic functional metrics recorded in simulated heatwave in situ experiment.

|  | **Functional Richness** | | | **Functional Evenness** | | | **Functional Dispersion** | | | **Functional Redundancy** | | |
| --- | --- | --- | --- | --- | --- | --- | --- | --- | --- | --- | --- | --- |
| **Treatment** | **Mean** | **Max** | **Min** | **Mean** | **Max** | **Min** | **Mean** | **Max** | **Min** | **Mean** | **Max** | **Min** |
| **Control** | 140.703 | 947.626 | 0.008 | 0.545 | 0.740 | 0.740 | 3.455 | 5.304 | 1.450 | 11.302 | 13.560 | 9.554 |
| **Long** | 182.776 | 881.950 | 0.168 | 0.514 | 0.605 | 0.605 | 4.037 | 4.995 | 2.466 | 11.405 | 12.616 | 10.215 |
| **Short** | 45.566 | 686.965 | 0.020 | 0.552 | 0.730 | 0.730 | 3.735 | 4.974 | 1.882 | 11.236 | 13.353 | 9.722 |
