# Supplementary material for: Redundancy of macrobenthic functional traits boosts resilience to a simulated heatwave
Source: PLoS One. 2026 Jan 12;21(1):e0340819. doi: 10.1371/journal.pone.0340819 (PMC12795362; doi:10.1371/journal.pone.0340819)
Supplement: Attachment — Submitted filename: Review assignment for PONE-D-25-44021.pdf [file pone.0340819.s008.pdf]

Review assignment for PONE-D-25-44021: Resilience of macrobenthic functional traits to simulate heatwaves

First, I would like to thank the authors for this interesting work, which I believe can make an excellent contribution to the study of functional diversity and the application of its metrics, which are necessary for a better understanding of how ecosystems function in the context of global climate change. Below is a general comment on the manuscript, followed by comments on each section.

Overall, the study is well conceptualised and structured, with a solid foundation in marine community ecology. The experimental concept is promising and provides valuable and interesting insights into how marine macrofaunal communities respond to the main effects of climate change, to which they are increasingly exposed. The experimental design is well constructed and balanced, supported by an adequate number of replicates, which gives the study good statistical representativeness and robustness.

However, in reporting and describing the study, several gaps were identified, particularly regarding sample collection, and an adequate description of the data processing for the calculation of the functional metrics, which are central to the study, was not provided.

As a result, it was not possible to consult the data (including means and standard deviations) obtained, as these were not clearly presented in either the main text or the supplementary materials. This makes it difficult to interpret the results, which are displayed only in the graphs, and also hinders comparison with other studies and a thorough evaluation of the work carried out.

For easier reading, I am also attaching a PDF file containing this information in this format, as well as a PDF file of the manuscript with the corresponding comments.

INTRODUCTION

Overall, the Introduction is well written, and the objectives of the study are clearly explained. However, it requires some additions. As the study mainly focuses on the "functional" aspects of the infaunal community, a brief, in-depth description and definition of functional diversity and its metrics is needed.

78: "Functional diversity also plays a critical role in resilience." This is true, but it would be useful to spend some time explaining what functional diversity is and how it can be measured. I suggest briefly describing the metrics used to estimate functional diversity (which is not straightforward) and providing some references. Describing these metrics may help readers, including non-specialists, to better understand your results.

94-95: "functional metrics (i.e., individual functional traits, functional indices)". It is not clear what type of indices you include under the definition of "individual functional traits". In particular, it is not clear from the initial reading whether this refers to the organism level or to a single functional trait. I assume it refers to a single functional trait. If so, I suggest changing "individual functional trait" to "single-trait indices" and "functional indices" to "multi-trait indices". "Single-trait indices" include, for example, the CWM that you mention later, while

"multi-trait indices" include "functional richness, evenness", etc. It would be more accurate to refer to these broader categories.

MATERIALS AND METHODS

The study and its objectives are promising, and it may provide new perspectives on understanding the responses of macrobenthic infaunal communities to more frequent events driven by global climate change. However, some aspects need clarification, from the experimental design and sample collection to data processing and analysis. Furthermore, for greater accuracy, it would be useful to include separate tables with the abundance matrix and species-trait matrix, perhaps in the supplementary materials. This would allow for a better understanding of the results.

Figure 1. Please check the caption and ensure that the letters correspond accurately to those indicated in the figure. Correct "e" to "d" in the figure.

111: Since you are discussing the OTCs, please refer directly to Figure 1d.

117-118: It is not clear whether the OTCs were removed during the night and at high tides. Please clarify.

119: For greater clarity, please specify that the "Control treatment" is without the OTCs.

129: Did you remove the temperature loggers from the control plots during the night and at high tide? How long is the tidal excursion? Did the logger measure temperature only in the top 1 cm of the sediment? If so, how can you be sure that the temperature also increases in the lower layers?

132-137: You mentioned the use of a PVC corer for macrofaunal sampling. How many cores did you collect per plot? Please specify. Furthermore, you used a PVC corer with a depth of 15 cm for macrofaunal sampling. Based on this, did you observe any sediment stratification? Did you consider all the macrofauna within the 15 cm, or did you collect it only from the surface layers? As you correctly mentioned in the introduction, some species may respond to thermal increases by moving within the sediment. Therefore, it would be interesting to observe this in the cores. Additionally, the procedure for macrofaunal sampling is not very clear. Please clarify: once collected, was the core rinsed and sieved through a 0.5 mm mesh?

160: Regarding Table 1, there is an inconsistency between the acronyms and the trait modalities for the traits "Living Habit", "Movement Method", and "Sediment Position". Please check and correct.

165-169: It is not clear how you calculated the four metrics, particularly which data you used. Generally, obtaining these metrics requires a combination of the species abundance matrix and the trait matrix (with fuzzy coding). For greater clarity, please revise this section to specify how you treated the data before calculating the metrics. This clarification would help readers understand the results obtained. If you used a different procedure, please specify it. Also, indicate whether you performed standardisation or transformation of the data.

174: Are the factors mentioned related to the simulated heatwaves (5 days and 7 days)? This is not clear. Please specify the factors considered and the levels for each factor.

172-178: This section is somewhat difficult to follow and may cause confusion. PERMANOVA was used to test the effects of MHWs on taxonomic and functional metrics, correct? If so, it is redundant to restate the factors. If not, it is unclear which factors are being tested. As mentioned above, please specify the factors being analysed. Additionally, the phrase "the Euclidean distance for the single macrobenthic metrics" is unclear; it is not specified which metrics are being referred to. I recommend clarifying this short paragraph to avoid misunderstandings regarding the statistical analysis performed.

183: Do "Functional trait data" refer to the combination of the macrofaunal data and trait "fuzzy coding assignation"?

184-187: This section is clearer than the previous one. I suggest making changes to the previous section, using this one as a starting point.

RESULTS

Overall, the results are well presented and consistent with the Methods and Materials section. However, I suggest further integration and more in-depth descriptions in the main text, as well as modifications to the plots. Generally, the choice of plot types is good, and they are easy to read, even for non-specialists. However, some plots need to be checked and adjusted to ensure consistency with the descriptions in the main text.

214-228: I suggest providing a supplementary table with the abundance and diversity data to support the interpretation of the boxplots. It may also be useful to describe in the main text the magnitude of these changes among the treatments (even if not significant in some cases), including values, as has already been done previously for temperature.

247-252: How does the community structure change among the treatments? I suggest providing a description of the community structure in all treatments and indicating which species or taxa drive the arrangement in the nMDS. The same applies to the functional trait composition. It is also possible to overlay as vectors the species that contribute most to these dissimilarities. Please take this into consideration. Otherwise, it must be specified which main differences are supported by the statistical analysis.

254–265: As in the previous comment, for greater comprehensiveness, it may be useful to provide at least the mean values and standard deviations of the multi-trait indices (FRic, FEve, etc.) to better understand the magnitude of the differences. I suggest adding a supplementary table with the means of all the descriptors considered (abundance, richness, diversity, FRic, FEve, etc.) to support the reading of the main text and the plots.

270-272: Figure 7. In the caption, the sentence "The colour scale indicates the magnitude of the correlations" is assumed to refer to the colour gradient bar on the right. Does it represent the interval of the Spearman correlation coefficient (rho (ρ) = +1, 0, -1)? If so, please specify this in the caption and also next to the scale bar. Furthermore, if this is the case, there appears to be an inconsistency between the main text and Figure 7. According to the main text, a significant negative Spearman correlation was found between the Control and the trait modality "burrower". In Figure 7, a negative correlation would be expected to be shown with a "blue" shade. Is the colour scale inverted? Please clarify this aspect to avoid misinterpretation of the results.

277-283: Figure 8. There is the same issue with the visualisation of the correlation. It appears that the interval is inverted: positive correlations are blue and negative correlations are red. Please ensure that the interval on the right matches exactly with the output of the level plot, as it currently appears inconsistent. As previously suggested, if the interval on the right (+1 / -1) corresponds to the Spearman coefficient (rho), please specify this next to the gradation scale in the plot and/or in the figure caption.

DISCUSSION

This section provides an overview of the study and its findings. However, although the arguments are valid and appropriate, they seem to enhance and reinforce the study and its findings only partially. The study required significant practical and interpretative effort, which I believe deserves greater recognition. While it is acknowledged that interpreting functional diversity metrics is not easy, if these metrics are used, it is necessary to provide a valid explanation of their selection and the type of information they convey. Only some of these metrics are explained, while others, which are also informative, are omitted. Furthermore, the arguments supporting 'functional' resilience are unconvincing because they rely solely on evidence of redundancy, which may result from bias in the selection of functional traits. I recommend that the authors supplement their argument by providing supporting data, for example in the form of tables. This would lend further weight to the arguments presented in this section and give due recognition to the work undertaken.

293-294: In my opinion, the findings related to the functional traits and metrics do not necessarily indicate resilience of the community but rather demonstrate redundancy in functional diversity during the experiment. Therefore, the sentence "however, functional traits and functional metrics were less affected, suggesting a degree of functional resilience" is problematic. Firstly, since Functional Evenness and Functional Dispersion changed significantly according to your results, this is inconsistent with your statement. Secondly, as I understand it, the experiment simulates a heatwave during low tide with relatively short exposure (4 hours per day, every day), alternating with high tide. Is this correct? Do you think this exposure is sufficient to expect changes in functional traits? Additionally, providing information on how many cores were collected from each plot each day would help to support this statement. Furthermore, a brief description of the community in the "Results" section could also help to predict the response to the heatwave experiment.

Furthermore, in selecting the functional trait, I notice some overlap between Living Habit and Movement Method, for example in the modalities "Crawler" and "Burrower". The choice of trait depends on the structure of the community, and sometimes different species may share the same functional trait. Since trait selection is arbitrary and there is no standard rule for how many or which traits to consider, have you tried considering only one of the two functional traits in question—either "Movement Method" or "Living Habit"? This might reduce overlap and redundancy. How can you explain that the observed redundancy is a response of the community to the HWs, rather than a redundancy resulting from the "over-expression" of the same trait due to overlap between trait modalities?

296–298: Does the increase in temperature refer only to the first centimetre (0–1 cm) of the sediment? Did you observe thermal increases below the first sediment layer as well? If so, as you correctly mentioned in the introduction, some species might move within the sediment. In

this case, documenting the effect of temperature increase could be challenging if the data refer only to the sediment surface.

330-346: What about the other indices, Functional Evenness and Functional Dispersion? According to your results, they change significantly between the control and treatments, as shown in the boxplot in Figure 6. It would be noteworthy to comment on their trends. Why does FDis increase significantly with exposure to the HW? The same question applies to FEve.
