# Supplementary material for: Redundancy of macrobenthic functional traits boosts resilience to a simulated heatwave
Source: PLoS One. 2026 Jan 12;21(1):e0340819. doi: 10.1371/journal.pone.0340819 (PMC12795362; doi:10.1371/journal.pone.0340819)
Supplement: Attachment — Submitted filename: PONE-D-25-44021_reviewed.pdf [file pone.0340819.s009.pdf]

PLOS ONE

Resilience of macrobenthic functional traits to simulated heatwave

--Manuscript Draft--

**Manuscript Number:**	PONE-D-25-44021
**Article Type:**	Research Article
**Full Title:**	Resilience of macrobenthic functional traits to simulated heatwave
**Short Title:**	Macrobenthic response to heat stress
**Corresponding Author:**	Orlando Lam-Gordillo<br>The New Zealand Institute for Earth Science<br>AUSTRALIA
**Keywords:**	Biological traits; climate change; heat stress; heat experiment; estuary; macrofauna
**Abstract:**	Climate change is affecting ecosystems worldwide. Ocean temperature is increasing leading to more frequent and intense extreme weather events such as heatwaves (HWs) and marine heatwaves (MHWs). These extreme temperature events can affect marine biodiversity, the relative success of different life history strategies, and the structure and function of whole ecological communities. Understanding the effects of HWs on marine communities and the mechanisms that enable marine communities to resist and recover from heat stress has become a research priority. Here, we investigated the effects of a simulated HW on macrobenthic communities and their functional traits in intertidal estuarine sediments using a novel in-situ seafloor warming experiment with two heatwave duration treatments. Our results revealed that the simulated HW influenced macrobenthic abundance and diversity, yet, functional traits and functional metrics were less affected. This suggests resilience to the simulated HW related to redundancy of macrobenthic functional traits. The idiosyncratic responses observed in our study reflects the complex and context-dependent macrobenthic responses to thermal extremes. Our findings suggest that maintaining functional trait diversity and redundancy could be key for boosting ecosystem resilience under increasing climatic extremes.
**Order of Authors:**	Orlando Lam-Gordillo, PhD
	Emily J. Douglas, PhD
	Sarah F. Hailes
	Andrew M. Lohrer, PhD
**Additional Information:**	
**Question**	**Response**
**Financial Disclosure**<br><br>Enter a financial disclosure statement that describes the sources of funding for the work included in this submission. Review the submission guidelines for detailed requirements. View published research articles from *PLOS ONE* for specific examples.<br><br>This statement is required for submission and **will appear in the published article** if the submission is accepted. Please make sure it is accurate.	Yes

Powered by Editorial Manager® and ProduXion Manager® from Aries Systems Corporation

**Funded studies**<br>Enter a statement with the following details:<br>• Initials of the authors who received each award<br>• Grant numbers awarded to each author<br>• The full name of each funder<br>• URL of each funder website<br>• Did the sponsors or funders play any role in the study design, data collection and analysis, decision to publish, or preparation of the manuscript?<br><br>Did you receive funding for this work?	
Please add funding details.<br>as follow-up to "**Financial Disclosure**<br><br>Enter a financial disclosure statement that describes the sources of funding for the work included in this submission. Review the submission guidelines for detailed requirements. View published research articles from *PLOS ONE* for specific examples.<br><br>This statement is required for submission and **will appear in the published article** if the submission is accepted. Please make sure it is accurate.<br><br>**Funded studies**<br>Enter a statement with the following details:<br>• Initials of the authors who received each award<br>• Grant numbers awarded to each author<br>• The full name of each funder<br>• URL of each funder website<br>• Did the sponsors or funders play any role in the study design, data collection and analysis, decision to publish, or preparation of the manuscript?<br><br>Did you receive funding for this work?"	This research was funded by the New Zealand Government's Strategic Science Investment Fund (SSIF) to the New Zealand Institute for Earth Sciences (ESNZ; FPRS2606).
Please select the country of your main research funder (please select carefully as in some cases this is used in fee calculation).	NEW ZEALAND - NZ

as follow-up to "**Financial Disclosure**<br><br>Enter a financial disclosure statement that describes the sources of funding for the work included in this submission. Review the submission guidelines for detailed requirements. View published research articles from *PLOS ONE* for specific examples.<br><br>This statement is required for submission and **will appear in the published article** if the submission is accepted. Please make sure it is accurate.<br><br>**Funded studies**<br>Enter a statement with the following details:<br>• Initials of the authors who received each award<br>• Grant numbers awarded to each author<br>• The full name of each funder<br>• URL of each funder website<br>• Did the sponsors or funders play any role in the study design, data collection and analysis, decision to publish, or preparation of the manuscript?<br><br>Did you receive funding for this work?"	
**Competing Interests**<br><br>Use the instructions below to enter a competing interest statement for this submission. On behalf of all authors, disclose any competing interests that could be perceived to bias this work—acknowledging all financial support and any other relevant financial or non-financial competing interests.<br><br>This statement is required for submission and **will appear in the published article** if the submission is accepted. Please make sure it is accurate and that any funding sources listed in your Funding Information later in the submission form are also declared in your Financial Disclosure statement.	The authors have declared that no competing interests exist.

View published research articles from *PLOS ONE* for specific examples.<br><br>**NO authors have competing interests**<br><br>Enter: *The authors have declared that no competing interests exist.*<br><br>**Authors with competing interests**<br><br>Enter competing interest details beginning with this statement:<br><br>*I have read the journal's policy and the authors of this manuscript have the following competing interests: [insert competing interests here]*<br><br>* typeset	
**Ethics Statement**<br><br>Enter an ethics statement for this submission. This statement is required if the study involved:<br><br>• Human participants<br>• Human specimens or tissue<br>• Vertebrate animals or cephalopods<br>• Vertebrate embryos or tissues<br>• Field research<br><br>Write "N/A" if the submission does not require an ethics statement.<br><br>General guidance is provided below. Consult the submission guidelines for detailed instructions. **Make sure that all information entered here is included in the Methods section of the manuscript.**	No permits were required for performing this research.

Powered by Editorial Manager® and ProduXion Manager® from Aries Systems Corporation

**Format for specific study types**<br><br>**Human Subject Research (involving human participants and/or tissue)**<br>• Give the name of the institutional review board or ethics committee that approved the study<br>• Include the approval number and/or a statement indicating approval of this research<br>• Indicate the form of consent obtained (written/oral) or the reason that consent was not obtained (e.g. the data were analyzed anonymously)<br><br>**Animal Research (involving vertebrate animals, embryos or tissues)**<br>• Provide the name of the Institutional Animal Care and Use Committee (IACUC) or other relevant ethics board that reviewed the study protocol, and indicate whether they approved this research or granted a formal waiver of ethical approval<br>• Include an approval number if one was obtained<br>• If the study involved *non-human primates*, add *additional details* about animal welfare and steps taken to ameliorate suffering<br>• If anesthesia, euthanasia, or any kind of animal sacrifice is part of the study, include briefly which substances and/or methods were applied<br><br>**Field Research**<br><br>Include the following details if this study involves the collection of plant, animal, or other materials from a natural setting:<br>• Field permit number<br>• Name of the institution or relevant body that granted permission	
**Data Availability**<br><br>Authors are required to make all data underlying the findings described fully available, without restriction, and from the time of publication. PLOS allows rare exceptions to address legal and ethical concerns. See the PLOS Data Policy and FAQ for detailed information.	Yes - all data are fully available without restriction

A Data Availability Statement describing where the data can be found is required at submission. Your answers to this question constitute the Data Availability Statement and **will be published in the article**, if accepted.<br><br>**Important:** Stating 'data available on request from the author' is not sufficient. If your data are only available upon request, select 'No' for the first question and explain your exceptional situation in the text box.<br><br>Do the authors confirm that all data underlying the findings described in their manuscript are fully available without restriction?	
**Describe where the data may be found in full sentences. If you are copying our sample text, replace any instances of XXX with the appropriate details.**<br><br>• If the data are **held or will be held in a public repository**, include URLs, accession numbers or DOIs. If this information will only be available after acceptance, indicate this by ticking the box below. For example: *All XXX files are available from the XXX database (accession number(s) XXX, XXX.*).<br>• If the data are all contained **within the manuscript and/or Supporting Information files**, enter the following: *All relevant data are within the manuscript and its Supporting Information files.*<br>• If neither of these applies but you are able to provide **details of access elsewhere**, with or without limitations, please do so. For example:<br><br>*Data cannot be shared publicly because of [XXX]. Data are available from the XXX Institutional Data Access / Ethics Committee (contact via XXX) for researchers who meet the criteria for access to confidential data.*<br><br>*The data underlying the results presented in the study are available from (include the name of the third party*	All relevant data are within the manuscript and its Supporting Information files.

and contact information or URL).<br>• This text is appropriate if the data are owned by a third party and authors do not have permission to share the data.<br><br>* typeset	
Additional data availability information:	

Manuscript

Click here to access/download;Manuscript;Manuscript_Macro_heatwave_v1

Resilience of macrobenthic functional traits to simulated heatwave

Orlando Lam-Gordillo[1*], Emily J. Douglas[1], Sarah Hailes[1], Andrew M. Lohrer[1]

[1]The New Zealand Institute for Earth Science, Hamilton, New Zealand.
*Corresponding author: orlando.lam-gordillo@niwa.co.nz

Abstract

Climate change is affecting ecosystems worldwide. Ocean temperature is increasing leading to more frequent and intense extreme weather events such as heatwaves (HWs) and marine heatwaves (MHWs). These extreme temperature events can affect marine biodiversity, the relative success of different life history strategies, and the structure and function of whole ecological communities. Understanding the effects of HWs on marine communities and the mechanisms that enable marine communities to resist and recover from heat stress has become a research priority. Here, we investigated the effects of a simulated HW on macrobenthic communities and their functional traits in intertidal estuarine sediments using a novel in-situ seafloor warming experiment with two heatwave duration treatments. Our results revealed that the simulated HW influenced macrobenthic abundance and diversity, yet, functional traits and functional metrics were less affected. This suggests resilience to the simulated HW related to redundancy of macrobenthic functional traits. The idiosyncratic responses observed in our study reflects the complex and context-dependent macrobenthic responses to thermal extremes. Our findings suggest that maintaining functional trait diversity and redundancy could be key for boosting ecosystem resilience under increasing climatic extremes.

Keywords: Biological traits, climate change, heat stress, heat experiment, estuary, macrofauna.

Introduction

Global climate is warming due to the increasing release of anthropogenic greenhouse gases to the atmosphere [1-3]. The increasing global temperature is leading to hotter summers and more frequent extreme climatic events such as heatwaves [1, 2, 4, 5]. In coastal ecosystems, marine heatwaves (MHWs) have already increased in intensity, frequency, and duration, with projections suggesting that this increasing pattern will continue in the future due to climate variability [2, 4, 6-8]. Intertidal coastal ecosystems are exposed to both marine and atmospheric heatwaves (HW) making them very vulnerable to climate change driven temperature anomalies [9].

Extreme temperature events including MHWs (i.e., prolongated periods – five or more days of sea surface temperatures above the 90th percentile) and HWs (i.e., prolonged periods of abnormally hot weather relative to the expected conditions) are likely to affect marine biodiversity, modifying the structure of communities, their distributional ranges, life history strategies, and survival [4, 6, 10-13]. These changes in biodiversity can have profound consequences to society, with losses of the essential ecosystem processes, functions, and services that these organisms and ecosystems provide to humankind [14-18]. Although several studies have assessed the effects of MHWs on biodiversity, focusing on kelp, macroalgae, coral reefs, and some marine invertebrates [e.g., 19, 20-23], less is known about the effects of HWs on intertidal macrobenthic assemblages and the factors affecting resistance and resilience to acute thermal stress in estuarine ecosystems.

Estuarine ecosystems are hotspots for biogeochemical cycling and biodiversity, and contribute to a wide range of ecosystem functions, services, and values (e.g., nutrient cycling, carbon storage, coastal protection, food production, cultural practices, amenity values) [24-26]. Estuarine macrobenthic fauna is generally well adapted to changes in temperature, including periodic natural cycles (e.g., daily temperature amplitude, tides, seasons), particularly intertidal species that are exposed to air during low tide periods [27-29]. Estuarine macrobenthic organisms are likely well adapted to cope with extreme weather events such as MHWs [30-32], however, negative impacts may occur when organisms face prolonged periods of temperature increase that exceed their tolerance levels [31, 33-35].

Marine heatwaves, alongside atmospheric heatwaves, can particularly affect the fitness of ectothermic estuarine organisms, whose physiological processes are highly sensitive to

temperature fluctuations [36-39]. Temperature fluctuations affects ectotherm metabolism [36, 37, 40], with repercussions on growth and reproductive rates, maximum body sizes, feeding behaviours, bioturbation, and other vital functions of macrobenthos [31, 35, 37, 41-43].

Understanding the mechanisms by which communities may resist and recover from HWs and MHWs has become a research priority. Some research has assessed the effects of HW on intertidal rocky shores [e.g., 44], yet limited information is available for estuarine soft sediments. In soft-sediment systems, analysis of functional traits is expanding our understanding of organismal responses to thermal extremes and the mechanisms underpinning community and functional change. Functional traits are properties of organisms that can be measured, usually at the individual organism level and used comparatively across species, facilitating the understanding of how organisms respond to environmental change [45, 46]. For example, mobile species or those capable of vertical migration within the sediment may avoid peak thermal exposure by relocating to cooler microhabitats, while deep-burrowing or tube-building taxa may experience buffered thermal regimes compared to surface dwellers [31, 32, 46]. Functional diversity also plays a critical role in resilience. For example, assemblages with high trait diversity are more likely to possess species that can maintain ecosystem functioning under stress, either through redundancy (i.e., multiple species sharing similar functional roles) or complementarity (i.e., different species compensating for different aspects of function) [47-49]. Identifying which traits boost resistance or facilitate recovery can improve predictions of ecosystem responses to climate extremes and inform conservation and management strategies.

In this study, we investigated the effects of a simulated heatwave during low tide on macrobenthic communities and their functional traits. We carried out and in-situ seafloor warming experiment using two short-term warming scenarios (5- and 7-days duration). We aimed to (i) evaluate changes in macrobenthic communities, their functional traits, and functional diversity in different warming scenarios, (ii) investigate the relationship between thermal stress (simulated heatwave) and macrobenthic communities, functional traits, and functional diversity, and (iii) elucidate if the variability in responses of functional traits and functional metrics to thermal stress confers resilience to heatwaves. We hypothesised that macrobenthic taxonomical (i.e., abundance, richness, diversity) and functional metrics (i.e., individual functional traits, functional indices) would decrease with a longer duration of

warming (7 days, relative to 5 days) as temperature is a natural trigger of essential ecological processes with profound consequences if the thermal tolerance of the organisms is exceeded.

Methods

Study area

The experiment was carried out in Waihī Estuary, in the Bay of Plenty region of the North Island of New Zealand (Figure 1). Waihī Estuary is a tidal lagoon type estuary (tidal range 1.7 m) that is permanently open to the sea and dominated by intertidal soft-sediment flats (57% of the estuary). The selection of the experimental site (Figure 1b-c) was based on a previous habitat mapping in the estuary [50] showing moderate muddy conditions (silt-clay: 26.65%), which represents the overall condition of estuaries across New Zealand.

Experimental design and set up

The experiment was conducted over a seven-day period in austral summer (17-23 February 2024). We deployed purpose-built Open Topped Chambers (OTCs) to the tidal flat in our experimental site (Figure 1) at the start of each low tide period during seven days, following the protocols described by Douglas, Lam-Gordillo (51) and Lam-Gordillo, Douglas (34). In brief, the cone-shaped OTCs (80cm base diameter, 30 cm top diameter) were constructed from transparent polycarbonate plastic (1.5 mm thickness) to promote the passive heating of enclosed sediments. OTCs were deployed (bottom edge pushed 1cm into the sediment surface) for four hours during daytime low tides for a period five and seven days (Short and Long duration treatments, respectively). Experimental plots were unenclosed and 'untreated' at night and during every high tide period during the experiment. Ten replicate plots per treatment (Control, Short, Long; total n= 30) were established and arranged using a randomised block design which covered an area of 10 x 60 m with 5 m separation between each plot. Our design enabled statistical analysis based on the factor "Treatment" (three levels: Control, Short, Long).

Data collection

Environmental data

Temperatures (°C) inside all control and treatment plots were measured using temperature loggers (EnvLoggers, Electric Blue CRL) located 1 cm below the sediment surface on wooden stakes (Figure 1d). Temperatures were recorded every minute and logged in all plots (Control, Long, Short) during the experiment.

Macrobenthic fauna sampling

Cores of sediment for the sampling of macrobenthos were collected from each of the control and experimental plots at the conclusion of each of the experimental treatment periods: Short treatment (Day 5) and Long treatment (Day 7). Macrobenthic fauna were collected by hand using a PVC corer (13 cm diameter by 15 cm depth), sieved through a 0.5 mm mesh size, and preserved in 70% isopropanol. Benthic macrofauna were sorted, identified to the lowest possible taxonomic level following standardised protocols [52], and counted.

Data analysis

Data recorded by the temperature loggers were analysed using the 'myClim' package [53] in R software [54]. Temperature data were first trimmed to only include times when the OTCs were deployed, and the resulting one-minute observations from each OTC period were averaged, providing one representative mean sediment temperature value per day per plot. In addition, we also calculated the minimum and maximum sediment temperature recorded during the times when the OTCs were deployed.

To test for differences in temperature between treatments, univariate PERMutational ANalysis Of VAriance (PERMANOVA) tests were conducted. PERMANOVA tests were based on Euclidean distance for the single variables (i.e., mean, minimum, and maximum temperature), permutation of residuals under a reduced model, Type III sums of squares, and 9999 permutations. In addition, multiple contrasts (pair-wise tests with 9999 permutations) were conducted if the fixed factor was significant in the main tests. Boxplots were constructed using the package 'ggpubr' [55] in R software [54].

To evaluate the effects of the simulated HWs on macrobenthic fauna, traditional and functional metrics were calculated using the packages 'vegan' [56] and 'FD' [57] in R software [54]. Traditional diversity metrics included taxonomic richness (S), Shannon-Wiener diversity (H', log e), and the total abundance (N) of macrofauna at each control and experimental plot. Functional metrics were calculated based on the selection of a suite of six functional traits and 27 traits modalities (Table 1). The functional traits selected describe behavioural, morphological, and physiological characteristics of the organisms that can be influenced by thermal stress. Trait information was retrieved from The New Zealand Trait Database for shallow-water marine benthic invertebrates – NZTD [58]. The NZTD uses a

fuzzy coding procedure, assigning scores from 0 to 1 to each taxon, with 0 being no affinity and 1 being high affinity to a trait [for details see 58]. Four functional metrics were calculated including Functional Richness (FRic), Fucntional Evenness (FEve), Fucntional Dispersion (FDis), and Functional Redundancy (FR – calculated as the ratio between RaoQ entropy and H'). In addition, Community-level Weighted Means of trait values (CWM) were calculated to compare trait expression across treatments. To facilitate trend visualisation, taxonomic and functional metrics were presented as boxplots constructed using the package 'ggpubr' [55], while trends in CWM trait values are showed as circular plots created using the package 'ggplot2' [59]. To investigate the effects of simulated MHWs in metrics (abundance, S, H', FRic, FEve, FDis, FR, and CWM) between treatments, PERMANOVA was used to analyse variation among factors, based on Euclidean distances for the single macrobenthic metrics, permutation of residuals under a reduced model, sums of squares type III, and 9999 permutations [60]. In addition, multiple pair-wise tests were conducted if the fixed factor (treatment) was significant to identify which groupings contributed to differences from PERMANOVA main tests.

To assess community and functional traits structure differences between treatments, bootstrapped non-Metric Multidimensional Scaling (nMDS) sample ordination plots were created based on a Bray-Curtis similarity resemblance applied to the macrobenthic abundance and functional trait data [60]. To test for differences in community structure for both macrobenthic and trait data, PERMANOVA tests were performed using fourth root transformed macrofauna data, Bray Curtis Similarities, permutation of residuals under a reduced model, sums of squares type III and 9999 permutations [60]. Pairwise tests were also conducted if main effects were significant.

Pearson correlations were performed to investigate the relationships between CWM traits, macrobenthic taxonomic and functional metrics and simulated MHWs conditions (mean, minimum, and maximum temperature) using the package 'corrplot' [61], and results were visualised through level plots constructed using the package 'lattice' [62].

Results

Simulated marine heatwave: intertidal sediment warming

Open Top Chambers increased the temperature of the sediments in the experimental plots compared to control plots successfully simulating a HW (Figure 2). Mean sediment temperature recorded in the experimental plots (i.e., Short and Long treatments) during the 7-

day period ranged from 24 °C to 34 °C, while in the control plots ranged from 23 °C to 28 °C (Figure 2a). Mean sediment temperature was significantly different among treatments (PERMANOVA $p<0.05$; Figure 2a). Pairwise post-hoc tests revealed that both Short and Long treatments were significantly warmer than Controls ($p<0.05$; Figure 2a). Minimum temperature across the experimental plots ranged from 20 °C to 30 °C and in the control plots 20 °C to 27 °C, while the maximum temperature recorded ranged from 24 °C and 36 °C in the experimental plots and from 24 °C to 30 °C in the control plots (Figure 2a). Similar to mean sediment temperature, there were significant differences in minimum and maximum sediment temperature among treatments ($p<0.05$; Figure 2a). Pairwise tests again showed that both Short and Long treatments were significantly warmer than Controls ($p<0.05$; Figure 2a). The overall temperature (i.e., combining the temperatures from all the experimental days recorded during OTC incubations) showed similar patterns to the daily temperatures (Figure 2b). Mean, minimum, and maximum sediment temperature differed significantly between Controls and Long and Short treatments (PERMANOVA $p<0.05$; Figure 2b), showing higher temperature in the experimental plots compared to the Controls.

Effects of simulated heatwaves on macrobenthic and functional metrics

In total, 8,769 macrobenthic organisms were collected and 25 macrobenthic taxa were identified in our in-situ warming experiment (Table S1). Mean macrobenthic richness was higher in the Long treatment than the Control treatment, yet there was no significant difference across treatments (PERMANOVA $p>0.05$; Figure 3a). The highest macrobenthic mean total abundance was recorded in the Control treatment, while the lowest macrobenthic mean total abundance was found in the Short treatment (Figure 3b). Significant differences in macrobenthic abundance were found between treatments (PERMANOVA $p<0.05$; Figure 3b), revealing significantly lower abundance in the Short and Long treatments compared to the Control treatment ($p<0.05$; Figure 3b). The highest mean macrobenthic diversity was found in the Long treatment and the lowest macrobenthic diversity in the Control treatment (Figure 3c). Significant differences in macrobenthic diversity across treatments were also found (PERMANOVA $p<0.05$; Figure 3c), which followed the same pattern as macrobenthic abundance, showing significantly higher diversity in Short and Long treatments compared to Control treatment ($p<0.05$; Figure 3b).

Across all treatments, the most expressed functional trait modalities, based on community-level weighted means (CWM) analyses of trait values, were surface modifiers (Bioturbation;

contribution: 52 %), medium body size (5–20 mm) (contribution: 100 %), filter/suspension feeders (Feeding mode; contribution: 74 %), tube dwelling (Living habit; contribution: 75 %), burrower (Movement method; contribution: 53 %), and organisms positioned in the surface shallow (<3 cm) of the sediment (Living position; contribution: 50 %) (Figure 4). Significant differences in functional traits and their modalities (as CWM) across treatments were identified (Table 2; Figure 4). Most of the significant differences were found in the functional trait 'Feeding mode', for example, suspension feeders were significantly higher in the Control treatment compared to the Short and Long treatment, while predators and scavengers were significantly lower in the Control treatment compared to the Short and Long treatment (Table 2; Figure 4). Other significant differences in CWM trait modalities were identified in 'Bioturbation', 'Living habit', and 'Sediment position' and mostly between the Control and Long treatment (Table 2; Figure 4).

Significant differences in community and functional traits composition were detected between treatments (PERMANOVA $p<0.05$; Figure 5). The bootstrapped nMDS plot for macrobenthic community structure revealed two main groups (Figure 5a). These groups were significantly different in community structure, differentiating the Control treatment from the Short and Long treatments ($p<0.05$, Figure 5a). Functional traits composition followed the same pattern as the community structure, revealing significant differences in functional trait structure between the Control treatment and the Short and Long treatments ($p<0.05$; Figure 5b).

Macrobenthic Functional Richness (FRic) was homogenous across treatments and no significant differences were found between treatments (PERMANOVA $p>0.05$; Figure 6a). The highest mean total FRic was recorded at the Long treatment, while the lowest mean total was identified at the Short treatment (Figure 6a). Significant differences in macrobenthic Functional Evenness (FEve) between treatments were identified (PERMANOVA $p<0.05$), and pairwise test revealed significant differences between the Short and Long treatments but no difference between Control and OTC treatments ($p<0.05$; Figure 6b). Macrobenthic Functional Dispersion (FDis) was significantly different across treatments (PERMANOVA $p<0.05$) with significantly higher values in Short and Long treatments compared to Control treatment ($p<0.05$; Figure 6c). Macrobenthic Functional Redundancy (FR) followed a similar pattern as FRic with no significant differences between treatments (PERMANOVA $p>0.05$; Figure 6d).

Correlations between CWM trait modalities and the mean, minimum, and maximum temperature recorded during the in-situ warming experiment showed idiosyncratic positive and negative correlations (Figure 7). For example, in Control treatment, significant negative correlations ($p<0.05$) were found between the trait modality 'burrower' and the mean, minimum, and maximum temperature, relationships that were not evident in the OTC treatments. In Short and Long treatments, 'bioirrigator', 'scavenger', and 'predator' trait modalities were negatively correlated to temperature, compared with weak or positive relationships in the Controls. In general, the long treatment showed more negative correlations compared to Control and Short treatments. All correlations, except for 'burrower' in Control treatment, were not statistically significant ($P>0.05$; Figure 7). Correlations between taxonomical and functional metrics and temperatures (i.e., mean, minimum, maximum) recorded in the in-situ experiment showed similar patterns across treatments (Figure 8). Significant positive relationships ($p<0.05$) was identified between the FEve and mean, minimum, and maximum temperature in Short treatment (Figure 8). Macrobenthic richness and abundance were negatively correlated to temperature, similar to FRic and FDis. Except from FEve, all other correlations were not statistically significant ($p>0.05$).

Discussion

Marine and atmospheric heatwaves are becoming more frequent, longer, and more intense globally. These extreme temperature events have repercussions when thermal tolerance of species is exceeded, causing for example mass mortalities and shifts in community structure of marine assemblages [4, 20, 21, 35, 63, 64]. In this study, we evaluated the effects of a simulated HW on macrobenthic communities and their functional traits using a novel in-situ intertidal warming experiment and two heatwave duration. Our findings revealed that simulated HW influenced the macrobenthic fauna taxonomical descriptors (i.e., abundance, diversity), however, functional traits and functional metrics were less affected, suggesting a degree of functional resilience to the simulated HW.

In contrast with our hypothesis, we found idiosyncratic responses to the simulated HW. Clear evidence of larger effects on macrobenthic fauna metrics (i.e., taxonomical and functional) with longer duration of warming was lacking. Our results demonstrated that simulated HW influenced macrobenthic community descriptors, particularly macrobenthic abundance and

diversity. These findings are consistent with previous studies reporting decreased abundance and altered species composition following natural or experimental heatwaves [35, 64-66]. For example, in our study most of the differences between the control and treatments were driving by the decrease on abundance of the amphipod *Paracorophium excavatum*. The abundance of this amphipod decreased almost half in Short and Long (~1700 organisms) treatments compared to the abundance recorded in the Control (~3000 organisms). These effects could be attributed to direct physiological stress or increased metabolic demand leading to energy deficits, reproductive failure, or mortality [34, 67-69]. However, again, consistent or stronger effects associated with longer heatwave duration were missing. This lack of clear duration-dependent effects suggests that the macrobenthic responses may be driven more by species-specific thermal tolerances or pre-existing environmental variability than by the heatwave duration alone [22, 31, 69, 70]. Temporal mismatch between peak temperature exposure and sensitive life stages, or behavioural adaptations such as vertical burrowing in the sediment, could further modulate exposure and susceptibility to the simulated HW [31, 32, 71, 72]. Diversity was significantly lower in Control treatment compared to Short and Long treatments, however, this effect was driven by the overall high abundance of dominant species (e.g., *Paracorophium excavatum*, Oligochaete, *Arthritica sp.*), reducing the evenness in abundance across species and explaining the lower diversity in the Control treatment [73, 74] rather than an effect due to the simulated HW.

Functional traits (CWM) varied across treatments with most of the significant differences found in the functional trait Feeding mode. For example, suspension feeders were significantly higher while predators and scavengers were significantly lower in the Control treatment compared to the Short and Long treatment. The elevated densities of scavengers in the Short and Long treatments likely reflect the cascading effects of thermal stress on benthic assemblages. Elevated temperatures can cause direct mortality or force organisms to emerge from the sediment, increasing their vulnerability to predation. Such events create feeding hotspots that can attract mobile predators and scavengers from surrounding areas [75, 76]. Similar scavenger aggregations have been documented following marine heatwaves and other disturbance events that elevate carrion availability on the seafloor [63, 64, 70, 77, 78]. Functional Richness and Functional Redundancy were less responsive to the simulated HW. This observation suggests a degree of resilience within the macrobenthic assemblages, even when community composition and abundance was altered. In other words, functional groups appear to remain well represented following heat exposure despite impacts on some

individual species within functional groups. Resilience based on functional traits could be explained by redundancy, where multiple taxa share similar trait modalities and therefor contribute similarly to ecosystem processes [22, 48, 49, 79, 80]. Our findings aligned with previous research suggesting weak or delayed effects of heatwaves on benthic ecosystem functioning [35, 64, 81]. Furthermore, species that differ taxonomically but share similar traits related to sediment mixing, feeding, or mobility may compensate for one another functionally. This redundancy could be acting as a buffer against thermal stress, maintaining processes such as bioturbation, bioirrigation, and nutrient cycling under changing environmental conditions [22, 31, 35, 64, 81, 82]. It is also plausible that certain heatwave-resistant taxa (characterised by traits such as deep burrowing behaviour, deposit feeders, or broad thermal tolerances) persisted across treatments and contributed largely to the functional profile of the assemblage [22, 31, 83, 84]. These traits may be acting as ecological filters, selecting for functionally important taxa under stress scenarios such as HWs.

The idiosyncratic nature of the responses observed in our study reflects the complex and context-dependent macrobenthic responses to thermal extremes. While temperature is a key driver of ectotherm organisms physiology, the response of macrobenthic communities is also shaped by multiple interacting environmental conditions and stressors such as anoxia, organic enrichment, eutrophication, and sediment characteristics [81, 85-89]. These factors could potentially either amplify or dampen the effects of thermal stress, depending on local habitat characteristics. Moreover, the response of functional traits and functional metrics to heat stress may not manifest uniformly across time and space. Traits related to feeding mode, bioturbation, and reproductive strategy can be slow to change or exhibit lag effects following a HW [22, 28, 29, 90].

Our study supports the idea that maintaining and enhancing functional trait diversity and redundancy could be key for boosting ecosystem resilience under increasing climatic extremes. Protecting habitat heterogeneity and limiting cumulative anthropogenic pressures that affect macrobenthic communities, their functional traits and ecological functions, may help preserve ecosystem functioning in a rapidly warming world.

Conclusion

We simulated an in-situ heatwave on estuarine intertidal sediments. Our results revealed variable responses in macrobenthic assemblages, with larger effects on macrobenthic

abundance and diversity, while in general, functional trait responses remained relatively stable during the warming experiment. These findings suggest that benthic communities may exhibit a degree of short-term functional resilience to heat stress, likely mediated by the filtering and redundancy of functional traits. However, the absence of clear duration effects and the idiosyncratic nature of responses highlight the complexity of predicting community and functional dynamics under future climate extremes. Continued use of in-situ experimentation and trait-based frameworks will be critical for improving our understanding of benthic ecosystem resilience in the face of climate change and increasing heatwaves.

References

1. Wong C. Earth just had its hottest year on record — climate change is to blame. Nature. 2023;623:674-5. doi: https://doi.org/10.1038/d41586-023-03523-3.
2. IPCC. Annex II: Glossary [Möller, V., R. van Diemen, J.B.R. Matthews, C. Méndez, S. Semenov, J.S. Fuglestvedt, A. Reisinger (eds.)]. In: Pörtner H-O, Roberts DC, Tignor M, Poloczanska ES, Mintenbeck K, Alegría A, et al., editors. Climate Change 2022: Impacts, Adaptation and Vulnerability Contribution of Working Group II to the Sixth Assessment Report of the Intergovernmental Panel on Climate Change: Cambridge, UK and New York, NY, USA; 2022. p. 2897–930.
3. Huguenin MF, Holmes RM, England MH. Drivers and distribution of global ocean heat uptake over the last half century. Nature Communications. 2022;13(1):4921. doi: 10.1038/s41467-022-32540-5.
4. Oliver ECJ, Donat MG, Burrows MT, Moore PJ, Smale DA, Alexander LV, et al. Longer and more frequent marine heatwaves over the past century. . Nat Commun. 2018. doi: https://doi.org/10.1038/s41467-018-03732-9.
5. Dong T, Zeng Z, Pan M, Wang D, Chen Y, Liang L, et al. Record-breaking 2023 marine heatwaves. Science. 2025;389(6758):369-74. doi: doi:10.1126/science.adr0910.
6. Prum P, Harris L, Gardner J. Widespread warming of Earth's estuaries. Limnology and Oceanography Letters. 2024;9(3):268-75. doi: https://doi.org/10.1002/lol2.10389.
7. Coumou D, Rahmstorf S. A decade of weather extremes. Nature Climate Change. 2012;2(7):491-6. doi: 10.1038/nclimate1452.
8. Oliver EC, Benthuysen JA, Darmaraki S, Donat MG, Hobday AJ, Holbrook NJ, et al. Marine heatwaves. Annual review of marine science. 2021;13(1):313-42.
9. Stillman JH, Amri AB, Holdreith JM, Hooper A, Leon RV, Pruett LR, et al. Ecophysiological responses to heat waves in the marine intertidal zone. Journal of Experimental Biology. 2025;228(2). doi: 10.1242/jeb.246503.
10. Campana SE, Stefánsdóttir RB, Jakobsdóttir K, Sólmundsson J. Shifting fish distributions in warming sub-Arctic oceans. Scientific Reports. 2020;10(1):16448. doi: 10.1038/s41598-020-73444-y.
11. Scanes E, Scanes PR, Ross PM. Climate change rapidly warms and acidifies Australian estuaries. Nature Communications. 2020;11(1):1803. doi: 10.1038/s41467-020-15550-z.
12. Hobday AJ, Alexander LV, Perkins SE, Smale DA, Straub SC, Oliver EC, et al. A hierarchical approach to defining marine heatwaves. Progress in oceanography. 2016;141:227-38.
13. Gomes DGE, Ruzicka JJ, Crozier LG, Huff DD, Brodeur RD, Stewart JD. Marine heatwaves disrupt ecosystem structure and function via altered food webs and energy flux. Nature Communications. 2024;15(1):1988. doi: 10.1038/s41467-024-46263-2.
14. Nagelkerken I, Connell SD. Global alteration of ocean ecosystem functioning due to increasing human CO2 emissions. Proceedings of the National Academy of Sciences. 2015;112(43):13272-7. doi: https://doi.org/10.1073/pnas.1510856112.

15. Mendoza-Portillo V, García-De León FJ, von der Heyden S. Responses of population structure and genomic diversity to climate change and fishing pressure in a pelagic fish. Global Change Biology. 2023;29:4107-25. doi: https://doi.org/10.1111/gcb.16732.
16. Virgen-Urcelay A, Donner SD. Increase in the extent of mass coral bleaching over the past half-century, based on an updated global database. PLOS ONE. 2023;18(2):e0281719. doi: 10.1371/journal.pone.0281719.
17. Queirós JP, Borras-Chavez R, Friscourt N, Groß J, Lewis CB, Mergard G, et al. Southern Ocean food-webs and climate change: A short review and future directions. PLOS Climate. 2024;3(3):e0000358. doi: 10.1371/journal.pclm.0000358.
18. Huang M, Chen Y, Zhou W, Wei F. Assessing the response of marine fish communities to climate change and fishing. Conservation Biology. 2024;n/a(n/a):e14291. doi: https://doi.org/10.1111/cobi.14291.
19. Harris RM, Beaumont LJ, Vance TR, Tozer CR, Remenyi TA, Perkins-Kirkpatrick SE, et al. Biological responses to the press and pulse of climate trends and extreme events. Nature climate change. 2018;8(7):579-87.
20. Oliver EC, Benthuysen JA, Bindoff NL, Hobday AJ, Holbrook NJ, Mundy CN, et al. The unprecedented 2015/16 Tasman Sea marine heatwave. Nature communications. 2017;8(1):16101.
21. Wernberg T, Smale DA, Tuya F, Thomsen MS, Langlois TJ, De Bettignies T, et al. An extreme climatic event alters marine ecosystem structure in a global biodiversity hotspot. Nature Climate Change. 2013;3(1):78-82.
22. Leung JYS, Russell BD, Connell SD. Adaptive Responses of Marine Gastropods to Heatwaves. One Earth. 2019;1(3):374-81. doi: 10.1016/j.oneear.2019.10.025.
23. Maucieri DG, Kushner DJ, Bates AE. Enduring a Major Marine Heatwave: The Role of Local Cool Refugia and Kelp Forests in the Resilience of Marine Invertebrates. Diversity and Distributions. 2025;31(6):e70044. doi: https://doi.org/10.1111/ddi.70044.
24. Townsend M, Thrush SF. Ecosystem functioning, goods and services in the coastal environment. Auckland Regional Council Technical Report 2010:2010/033. 2010;(2010/033).
25. Thrush SF, Hewitt JE, Pilditch C, Norkko A. Ecology of Coastal Marine Sediments: Form, Function, and Change in the Anthropocene. Oxford. 2021. doi: https://doi.org/10.1093/oso/9780198804765.001.0001.
26. Cloern JE, Abreu PC, Carstensen J, Chauvaud L, Elmgren R, Grall J, et al. Human activities and climate variability drive fast-paced change across the world's estuarine–coastal ecosystems. Globlal Change Biology. 2016;22:513–29. doi: https://doi.org/10.1111/gcb.13059.
27. Johnson RG. Temperature variation in the infaunal environment of a sand flat. Limnology and oceanography. 1965;10(1):114-20.
28. Hewitt JE, Ellis JI, Thrush SF. Multiple stressors, nonlinear effects and the implications of climate change impacts on marine coastal ecosystems. Global Change Biology. 2016;22:2665-75. doi: doi: 10.1111/gcb.13176.
29. Lam-Gordillo O, Hewitt JE, Douglas EJ, Dudley BD, Holmes SJ, Hailes S, et al. Climatic, oceanic, freshwater, and local environmental drivers of New Zealand estuarine macroinvertebrates. Marine Environmental Research. 2024;197:106472. doi: https://doi.org/10.1016/j.marenvres.2024.106472.
30. Verdelhos T, Marques J, Anastácio P. Behavioral and mortality responses of the bivalves Scrobicularia plana and Cerastoderma edule to temperature, as indicator of climate change's potential impacts. Ecological Indicators. 2015;58:95-103.
31. Zhou Z, Bouma TJ, Fivash GS, Ysebaert T, van Ijzerloo L, van Dalen J, et al. Thermal stress affects bioturbators' burrowing behavior: A mesocosm experiment on common cockles (Cerastoderma edule). Science of The Total Environment. 2022;824:153621. doi: https://doi.org/10.1016/j.scitotenv.2022.153621.

32. Zhou Z, Steiner N, Fivash GS, Cozzoli F, Blok DB, van IJzerloo L, et al. Temporal dynamics of heatwaves are key drivers of sediment mixing by bioturbators. Limnology and Oceanography. 2023;68(5):1105-16. doi: https://doi.org/10.1002/lno.12332.
33. Whiteley NM, Mackenzie CL. 56Physiological responses of marine invertebrates to thermal stress. In: Solan M, Whiteley N, editors. Stressors in the Marine Environment: Physiological and ecological responses; societal implications: Oxford University Press; 2016. p. 0.
34. Lam-Gordillo O, Douglas EJ, Hailes SF, Cummings V, Lohrer AM. Effects of in situ experimental warming on metabolic expression in a soft sediment bivalve. Scientific Reports. 2025;15(1):1812. doi: 10.1038/s41598-025-86310-6.
35. Dolbeth M, Babe O, Costa DA, Mucha AP, Cardoso PG, Arenas F. Benthic estuarine communities' contribution to bioturbation under the experimental effect of marine heatwaves. Scientific Reports. 2021;11(1):11422. doi: 10.1038/s41598-021-90720-7.
36. Valente S, Colloca F. A dataset of thermal preferences for Mediterranean demersal and benthic macrofauna. Scientific Data. 2024;11(1):314. doi: 10.1038/s41597-024-03168-5.
37. de Luzinais VG, Gascuel D, Reygondeau G, Cheung WWL. Large potential impacts of marine heatwaves on ecosystem functioning. Global Change Biology. 2024;30(7):e17437. doi: https://doi.org/10.1111/gcb.17437.
38. Barriopedro D, García-Herrera R, Ordóñez C, Miralles DG, Salcedo-Sanz S. Heat Waves: Physical Understanding and Scientific Challenges. Reviews of Geophysics. 2023;61(2):e2022RG000780. doi: https://doi.org/10.1029/2022RG000780.
39. Harrington LJ, Frame D. Extreme heat in New Zealand: a synthesis. Climatic Change. 2022;174(1):2. doi: 10.1007/s10584-022-03427-7.
40. Brown JH, Gillooly JF, Allen AP, Savage VM, West GB. Toward a metabolic theory of ecology. Ecology. 2004;85(7):1771-89.
41. Payne NL, Smith JA, van der Meulen DE, Taylor MD, Watanabe YY, Takahashi A, et al. Temperature dependence of fish performance in the wild: links with species biogeography and physiological thermal tolerance. Functional Ecology. 2016;30(6):903-12.
42. Lear KO, Morgan DL, Whitty JM, Whitney NM, Byrnes EE, Beatty SJ, et al. Divergent field metabolic rates highlight the challenges of increasing temperatures and energy limitation in aquatic ectotherms. Oecologia. 2020;193(2):311-23.
43. Plumlee JD, Powers SP, Kimbro DL, Lehrter JC, Herrmann J, Mareska J. Increasing duration of heatwaves poses a threat to oyster sustainability in the Gulf of Mexico. Ecological Indicators. 2024;162:112015. doi: https://doi.org/10.1016/j.ecolind.2024.112015.
44. Helmuth B, Mieszkowska N, Moore P, Hawkins SJ. Living on the edge of two changing worlds: forecasting the responses of rocky intertidal ecosystems to climate change. Annu Rev Ecol Evol Syst. 2006;37(1):373-404.
45. Petchey OL, Gaston KJ. Functional diversity: back to basics and looking forward. Ecology Letters. 2006;9(6):741-58. Epub 2006/05/19. doi: https://doi.org/10.1111/j.1461-0248.2006.00924.x. PubMed PMID: 16706917.
46. Lam-Gordillo O, Baring R, Dittmann S. Ecosystem functioning and functional approaches on marine macrobenthic fauna: A research synthesis towards a global consensus. Ecological Indicators. 2020;115. doi: https://doi.org/10.1016/j.ecolind.2020.106379.
47. Lefcheck JS, Duffy JE. Multitrophic functional diversity predicts ecosystem functioning in experimental assemblages of estuarine consumers. Ecology. 2015;96(11):2973-83. doi: https://doi.org/10.1890/14-1977.1.
48. Mouillot D, Graham NA, Villeger S, Mason NW, Bellwood DR. A functional approach reveals community responses to disturbances. Trends Ecol Evol. 2013;28(3):167-77. Epub 2012/11/13. doi: 10.1016/j.tree.2012.10.004. PubMed PMID: 23141923.
49. Villéger S, Mason NWH, Mouillot D. NEW MULTIDIMENSIONAL FUNCTIONAL DIVERSITY INDICES FOR A MULTIFACETED FRAMEWORK IN FUNCTIONAL ECOLOGY. Ecology. 2008;89(8):2290-301. doi: https://doi.org/10.1890/07-1206.1.

50. Lam-Gordillo O, Hailes S, Carter K, Petersen GL, Ferries M, Salmond N, et al. Integrating rapid habitat mapping with community metrics and functional traits to assess estuarine ecological conditions: A New Zealand case study. Marine Pollution Bulletin. 2024;206:116717. doi: https://doi.org/10.1016/j.marpolbul.2024.116717.
51. Douglas E, Lam-Gordillo O, Hailes S, Lohrer A, Cummings V. Simulated heatwave alters intertidal estuary greenhouse gas fluxes: duration and degradation state determine response. Nature Communications. In review.
52. Greenfield BL, Hailes S, MacLean F, Peart R, Read GB, Marshall B, et al. Protocol for the Identification of Benthic Estuarine and Marine Macroinvertebrates: A guide for parataxonomists. C-SIG Coastal Taxonomic Resource Tool 40 p + appendix. 2023.
53. Man M, Kalčík V, Macek M, Brůna J, Hederová L, Wild J, et al.: Microclimate data handling and standardised analyses in R. Methods in Ecology and Evolution. 2023;14(9):2308-20. doi: https://doi.org/10.1111/2041-210X.14192.
54. R-Core-Team. R: A language and environment for statistical computing. R Foundation for Statistical Computing, Vienna, Austria. 2022. doi: https://www.R-project.org/.
55. Kassambara A. ggpubr: 'ggplot2' Based Publication Ready Plots. 2020. doi: https://CRAN.R-project.org/package=ggpubr.
56. Oksanen J, Kindt R, Legendre P, O'Hara B, Stevens MHH, Oksanen MJ, et al. The vegan package. Community ecology package. 2007;10(631-637):719.
57. Laliberté E, Legendre P, Shipley B. FD: measuring functional diversity from multiple traits, and other tools for functional ecology. . R package version 10-12. 2014. doi: https://cran.r-project.org/web/packages/FD/index.html.
58. Lam-Gordillo O, Lohrer AM, Hewitt J, Dittmann S. NZTD - The New Zealand Trait Database for shallow-water marine benthic invertebrates. Scientific Data. 2023;10(1):502. doi: 10.1038/s41597-023-02414-6.
59. Wickham H. ggplot2: Elegant Graphics for Data Analysis. Springer-Verlag New York. ISBN 978-3-319-24277-4. 2016. doi: https://ggplot2.tidyverse.org.
60. Anderson MJ, Gorley RN, Clarke KR. PERMANOVA+ for PRIMER: Guide to Software and Statistical Methods. Plymouth: PRIMER-E. 2008.
61. Wei T, Simko V, Levy M, Xie Y, Jin Y, Zemla J. Package 'corrplot'. Statistician. 2017;56(316):e24.
62. Sarkar D. Lattice: Multivariate Data Visualization with R. New York, NY: Springer. 2008. doi: 10.1007/978-0-387-75969-2.
63. Garrabou J, Coma R, Bensoussan N, Bally M, Chevaldonné P, Cigliano M, et al. Mass mortality in Northwestern Mediterranean rocky benthic communities: effects of the 2003 heat wave. Global change biology. 2009;15(5):1090-103.
64. Pansch C, Scotti M, Barboza FR, Al-Janabi B, Brakel J, Briski E, et al. Heat waves and their significance for a temperate benthic community: A near-natural experimental approach. Global Change Biology. 2018;24(9):4357-67. doi: https://doi.org/10.1111/gcb.14282.
65. Grilo TF, Cardoso PG, Dolbeth M, Bordalo MD, Pardal MA. Effects of extreme climate events on the macrobenthic communities' structure and functioning of a temperate estuary. Mar Pollut Bull. 2011;62(2):303-11. Epub 2010/11/13. doi: 10.1016/j.marpolbul.2010.10.010. PubMed PMID: 21071045.
66. Pitacco V, Mistri M, Munari C. Long-term variability of macrobenthic community in a shallow coastal lagoon (Valli di Comacchio, northern Adriatic): Is community resistant to climate changes? Marine Environmental Research. 2018;137:73-87. doi: https://doi.org/10.1016/j.marenvres.2018.02.026.
67. Harley CDG, Randall Hughes A, Hultgren KM, Miner BG, Sorte CJB, Thornber CS, et al. The impacts of climate change in coastal marine systems. Ecology Letters. 2006;9(2):228-41. doi: https://doi.org/10.1111/j.1461-0248.2005.00871.x.

68. Stillman JH. Heat Waves, the New Normal: Summertime Temperature Extremes Will Impact Animals, Ecosystems, and Human Communities. Physiology. 2019;34(2):86-100. doi: 10.1152/physiol.00040.2018. PubMed PMID: 30724132.
69. Smale DA, Wernberg T, Vanderklift MA. Regional-scale variability in the response of benthic macroinvertebrate assemblages to a marine heatwave. Marine Ecology Progress Series. 2017;568:17-30.
70. Smale DA, Wernberg T, Oliver ECJ, Thomsen M, Harvey BP, Straub SC, et al. Marine heatwaves threaten global biodiversity and the provision of ecosystem services. Nature Climate Change. 2019;9(4):306-12. doi: 10.1038/s41558-019-0412-1.
71. Kristensen E, Penha-Lopes G, Delefosse M, Valdemarsen T, Quintana CO, Banta GT. What is bioturbation? The need for a precise definition for fauna in aquatic sciences. Marine Ecology Progress Series. 2012;446:285-302. doi: https://doi.org/10.3354/meps09506.
72. Queiros AM, Birchenough SN, Bremner J, Godbold JA, Parker RE, Romero-Ramirez A, et al. A bioturbation classification of European marine infaunal invertebrates. Ecology and Evolution. 2013;3(11):3958-85. Epub 2013/11/08. doi: https://doi.org/10.1002/ece3.769. PubMed PMID: 24198953; PubMed Central PMCID: PMCPMC3810888.
73. Gilbert B, Turkington R, Srivastava DS. Dominant species and diversity: linking relative abundance to controls of species establishment. The American Naturalist. 2009;174(6):850-62.
74. Winfree R, W. Fox J, Williams NM, Reilly JR, Cariveau DP. Abundance of common species, not species richness, drives delivery of a real-world ecosystem service. Ecology Letters. 2015;18(7):626-35. doi: https://doi.org/10.1111/ele.12424.
75. Beasley JC, Olson Z, DeVault T. Carrion cycling in food webs: comparisons among terrestrial and marine ecosystems. Oikos. 2012;121(7):1021-6.
76. Schlacher TA, Strydom S, Connolly RM. Multiple scavengers respond rapidly to pulsed carrion resources at the land–ocean interface. Acta Oecologica. 2013;48:7-12.
77. Sorte CJ, Pandori LL, Cai S, Davis KA. Predicting persistence in benthic marine species with complex life cycles: linking dispersal dynamics to redistribution potential and thermal tolerance limits. Marine Biology. 2018;165(1):20.
78. Rivetti I, Fraschetti S, Lionello P, Zambianchi E, Boero F. Global warming and mass mortalities of benthic invertebrates in the Mediterranean Sea. PloS one. 2014;9(12):e115655.
79. Gladstone-Gallagher RV, Pilditch CA, Stephenson F, Thrush SF. Linking Traits across Ecological Scales Determines Functional Resilience. Trends Ecol Evol. 2019;34(12):1080-91. Epub 2019/08/20. doi: 10.1016/j.tree.2019.07.010. PubMed PMID: 31422892.
80. Mouchet MA, Villéger S, Mason NWH, Mouillot D. Functional diversity measures: an overview of their redundancy and their ability to discriminate community assembly rules. Functional Ecology. 2010;24(4):867-76. doi: 10.1111/j.1365-2435.2010.01695.x.
81. Kauppi L, Villnäs A. Marine heatwaves of differing intensities lead to distinct patterns in seafloor functioning. Proceedings of the Royal Society B. 2022;289(1986):20221159.
82. Solan M, Cardinale BJ, Downing AL, Engelhardt KAM, Ruesink JL, Srivastava DS. Extinction and Ecosystem Function in the Marine Benthos. Science. 2004;306(5699):1177-80. doi: doi:10.1126/science.1103960.
83. Violle C, Navas M-L, Vile D, Kazakou E, Fortunel C, Hummel I, et al. Let the concept of trait be functional! Oikos. 2007;116(5):882-92. doi: https://doi.org/10.1111/j.0030-1299.2007.15559.x.
84. Staniek MA, Pansch C, Shama LN, Mehler K, Steinmann A, Middelburg JJ, et al. Heatwave intensity drives eco - physiological responses in infaunal bivalves: A mesocosm experiment. Limnology and Oceanography. 2025.
85. Villnäs A, Janas U, Josefson AB, Kendzierska H, Nygård H, Norkko J, et al. Changes in macrofaunal biological traits across estuarine gradients: implications for the coastal nutrient filter. Marine Ecology Progress Series. 2019;622:31-48. doi: 10.3354/meps13008.
86. Villnas A, Norkko J, Lukkari K, Hewitt J, Norkko A. Consequences of increasing hypoxic disturbance on benthic communities and ecosystem functioning. PLoS One. 2012;7(10):e44920.

Epub 2012/10/24. doi: 10.1371/journal.pone.0044920. PubMed PMID: 23091592; PubMed Central PMCID: PMCPMC3473027.
87. Douglas EJ, Hewitt J, Lohrer AM, Stephenson F. Changing intra- and interspecific interactions across sedimentary and environmental stress gradients. Ecosphere. 2023;14:e4373. doi: https://doi.org/10.1002/ecs2.4373.
88. Douglas EJ, Lohrer AM, Pilditch CA. Biodiversity breakpoints along stress gradients in estuaries and associated shifts in ecosystem interactions. Scientific Reports. 2019;9(1):17567. Epub 2019/11/28. doi: https://doi.org/10.1038/s41598-019-54192-0. PubMed PMID: 31772300; PubMed Central PMCID: PMCPMC6879482.
89. Douglas EJ, Pilditch CA, Lohrer AM, Savage C, Schipper LA, Thrush SF. Sedimentary Environment Influences Ecosystem Response to Nutrient Enrichment. Estuaries and Coasts. 2018;41:1994–2008. doi: https://doi.org/10.1007/s12237-018-0416-5.
90. Svensson JR, Lindegarth M, Pavia H. Equal rates of disturbance cause different patterns of diversity. Ecology. 2009;90(2):496-505.

Acknowledgements

We would like to thank Te Rūnanga o Ngāti Whakahemo and Professor Kura Paul-Burke (Waikato University) for inviting and warmly welcoming ESNZ team on to the Pukehina Marae and encouraging us to carry out the fieldwork and experimentation in Waihī estuary. We also thank the ESNZ Marine Ecology team in Hamilton, New Zealand for the processing of sediment and macrobenthic samples.

Author contributions

OLG and EJD conceived the study and led data compilation and manuscript preparation. OLG conducted the statistical analysis. OLG, EJD, SH, and AML conducted the fieldwork and experiment to generate the data. SH contributed to manuscript preparation. AML conceived provided statistical advice, and contributed to manuscript preparation and finalisation.

Data availability statement

All the data used is presented in the article and supplementary materials.

Funding

This research was funded by the New Zealand Government's Strategic Science Investment Fund (SSIF) to the New Zealand Institute for Earth Sciences (ESNZ; FPRS2606).

Conflict of interest

No potential conflict of interest was reported by the authors.

List of Tables

Table 1. List of the functional traits and traits-modalities selected based on their response to extreme weather events [modified from 58]. Acronyms are used in Figure 4.

Functional trait	Trait modalities	Acronyms
Bioturbator	Biodiffusor	Bdiff
	Bioirrigator	Birrig
	No bioturbation	Nbio
	Surface modifier	Sumo
Body size	Large (20mm)	Lar
	Medium (5-20mm)	Med
	Small (0.5-5mm)	Sma
Feeding mode	Deposit feeder	Defe
	Filter suspension	Fisus
	Grazer/scraper	Graz/Sc
	Predator	Pred
	Scavenger/opportunist	Scav
	Sub-surface deposit feeder	Ssdefe
Living habit	Burrower	Att
	Crawler	Burr
	None	Free
	Swimmer	Parc
		Tubdw
Movement method	Burrower	Burr2
	Crawler	Craw
	None	Non
	Swimmer	Swim
Sediment position	Attached	
	Bentho-pelagic	Atta
	Epibenthic	Be-pel
	Deeper >3cm	Epi
	Surface shallow <3cm	Deep

Table 2. Test results from univariate one-way fixed factor PERMANOVA to compare functional trait modalities between treatments. Significant differences are shown in bold. NC: No computed.

**Fucntional trait**	**Trait modality**	**Control vs Short**		**Control vs Long**		**Short vs Long**	
		t	P (perm)	t	P (perm)	t	P (perm)
Bioturbation	Biodiffusor	0.6799	0.5058	1.9112	0.0777	1.1618	0.2644
	Bioirrigator	1.6882	0.1098	2.7380	**0.0057**	1.0558	0.3034
	No bioturbation	NC	NC	NC	NC	NC	NC
	Surface modifier	0.0430	0.9678	0.5249	0.6088	0.6538	0.5199
Body size	Large (>20mm)	NC	NC	NC	NC	NC	NC
	Medium (5-20mm)	NC	NC	NC	NC	NC	NC
	Small (0.5-5mm)	NC	NC	NC	NC	NC	NC
Feeding mode	Deposit feeder	1.7449	0.0972	2.6990	**0.0100**	1.1523	0.2677
	Filter suspension	2.4124	**0.0212**	4.0076	**0.0002**	1.3219	0.1970
	Grazer / Scraper	0.6537	0.5251	2.3037	**0.0360**	1.9252	0.0726
	Predator	2.8357	**0.0061**	3.7977	**0.0014**	1.1233	0.2751
	Scavenger / Opportunist	2.6013	**0.0072**	4.2949	**0.0002**	1.2788	0.2205
	Sub surface deposit feeder	1.1814	0.2736	1.2618	0.2311	0.0583	0.9469
Living habit	Attached	NC	NC	NC	NC	NC	NC
	Burrower	1.3723	0.1888	3.0606	**0.0088**	1.3242	0.2046
	Free living / Surface crawler	2.2810	**0.0298**	3.8364	**0.0017**	0.7706	0.4463
	Parasite / Commensal	0.9461	0.3707	1.0646	0.3224	0.0534	0.9662
	Tube dwelling	1.8337	0.0792	3.6780	**0.0007**	1.1752	0.2611
Movement method	Burrower	0.9731	0.3389	0.6805	0.5201	0.0579	0.9534
	Crawler	0.9346	0.3665	0.5573	0.5865	0.0993	0.9239
	None	NC	NC	NC	NC	NC	NC
	Swimmer	0.3760	1.0000	0.6236	1.0000	0.5566	0.5563
Sediment position	Attached	NC	NC	NC	NC	NC	NC
	Bentho-pelagic	0.5221	1.0000	0.6236	1.0000	0.2336	0.8609
	Epibenthic	2.4743	**0.0246**	2.6590	**0.0114**	0.6317	0.5348
	Deeper than 3cm	2.8336	**0.0100**	3.3729	**0.0018**	0.8777	0.3849
	Surface shallow <3cm	0.1455	0.8830	0.3285	0.7464	0.2677	0.8032

List of Figures Captions

Figure 1. Map of the study area showing a) the location of Waihī Estuary in New Zealand, and (b) the area of Waihī Estuary where (c) the experimental plots were located. e) Close-up view of OTC showing position of stake with temperature loggers attached.

Figure 2. Boxplots showing the sediment temperature (°C) recorded during the in-situ warming experiment. Column a) shows the mean, minimum, and maximum temperature recorded during each OTC period each day (seven consecutive days) for each treatment (Control, Short, Long, n = 10). Column b) shows the mean, minimum, and maximum temperature recorded during all OTC periods for the week (7 days) for each treatment (Control, Short, Long, n = 70). Note the lower temperatures and smaller control-treatment differences on days 6 and 7 due to the arrival of cooler, cloudier weather. Thick lines = median, dots = outliers.

Figure 3. Boxplots showing the macrobenthic (a) richness (number of taxa), (b) abundance (total number of individuals), and (c) diversity (H') across treatments (Control, Short, Long) in Waihī Estuary. Thick lines = median, dots = outliers. Significant differences $p<0.05$ between treatments are shown as "a, b".

Figure 4. Community-weighted means (CWM) of functional trait-modalities expression. Scale represents the proportional contribution (each concentric line represents 0.25) to CWM recorded in (a) Control, (b) Short, and (c) Long treatments in Waihī Estuary. Trait modalities labels (acronyms) are defined in Table 1.

Figure 5. Bootstrapped Metric Multidimensional Scaling (MDS) ordination plot showing the macrobenthic (a) community structure and (b) trait composition across treatments in Waihī Estuary. Symbols close together in ordination space indicate greater similarity in multivariate composition.

Figure 6. Boxplots showing the macrobenthic (a) Functional Richness, (b) Functional Evenness, (c) Fucntional Dispersion, and (d) Functional Redundancy across treatments (Control, Short, Long) in Waihī Estuary. Thick lines = median, dots = outliers. Significant differences $p<0.05$ between treatments are shown as "a, b, c".

Figure 7. Level plot showing the spearman correlations between the mean, maximum, and minimum sediment temperature and functional traits in each of the treatments (Control, Short, Long). Fucntional traits – orange: Bioturbation, sky blue: Body size, green: Feeding mode, yellow: Living habit, blue: Movement method, vermilion: Sediment position. The colour scale indicates the magnitude of the correlations.

Figure 8. Level plot showing the spearman correlations between the mean, maximum, and minimum temperatures recorded at each of the treatments (Control, Short, Long) and the taxonomical and functional metrics. The colour scale indicates the magnitudes of the correlations. S: richness, N: abundance, H: diversity, FRic: Functional Richness, FEve: Functional Evenness, FDis: Fucntional Dispersion, FR: Functional Redundancy.

Figure 1

Click here to access/download;Figure;Figure1.tif

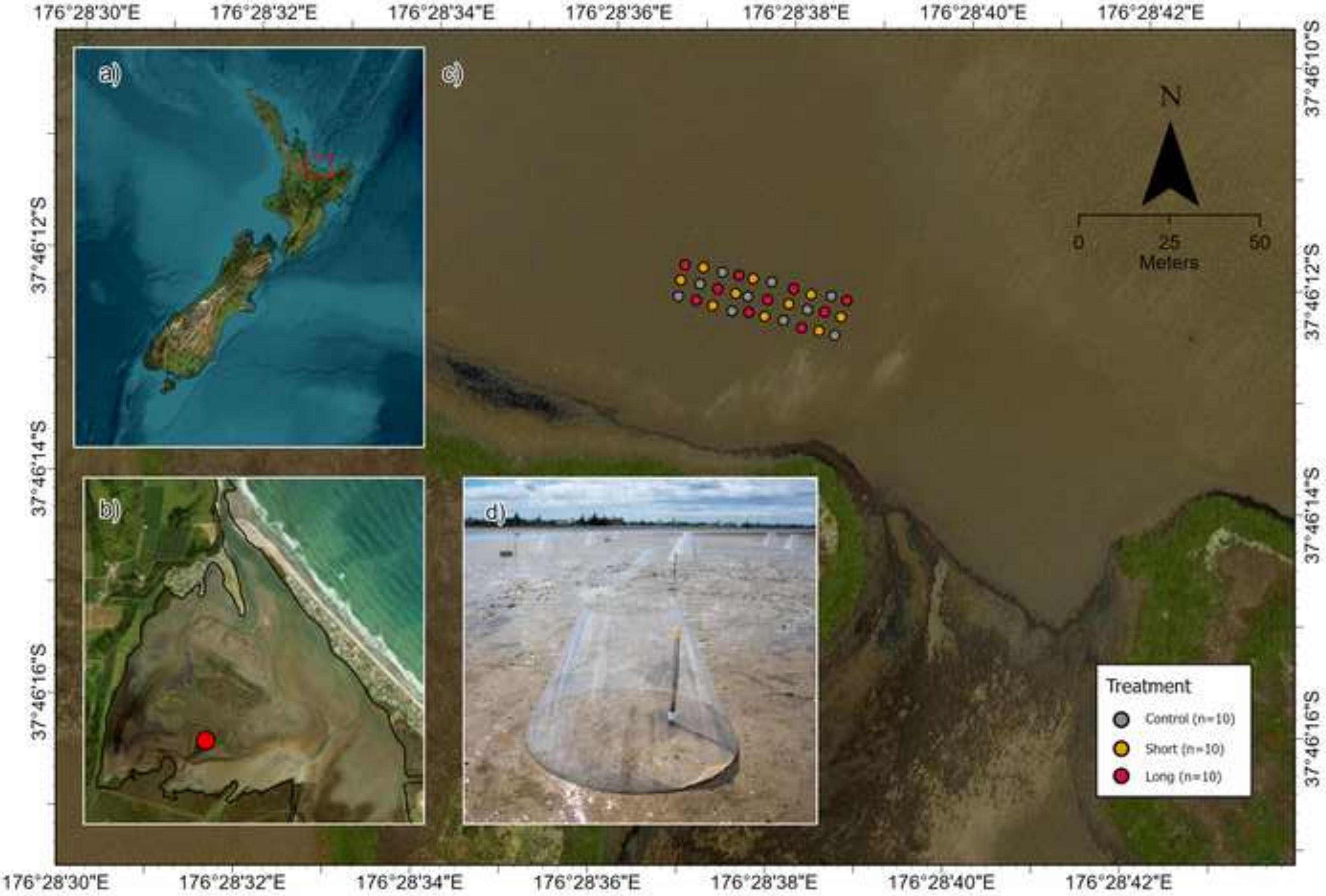

Figure 2

Click here to access/download;Figure;Figure2.tif

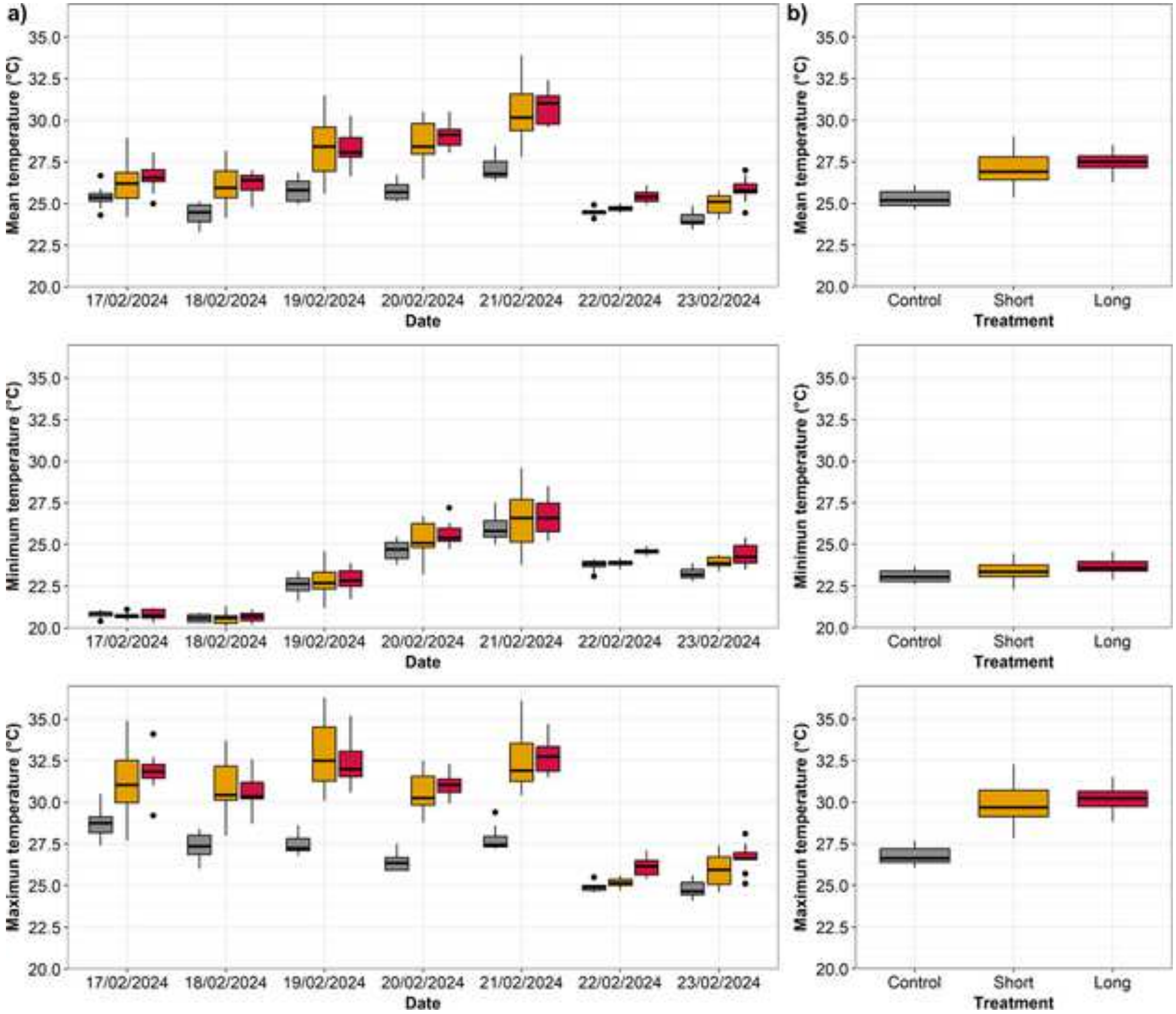

Figure3 Click here to access/download;Figure;Figure3.tif

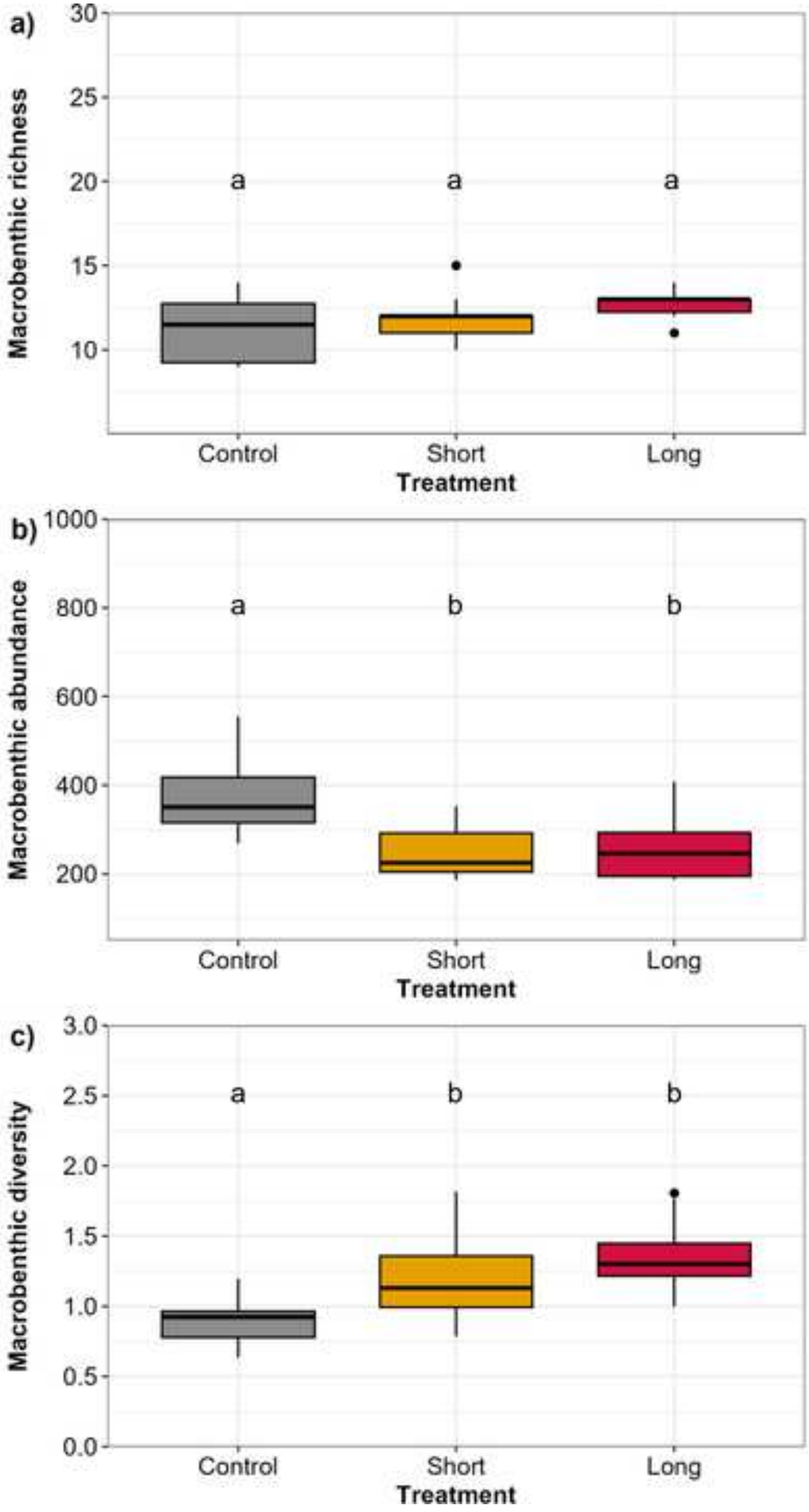

Figure4

Click here to access/download;Figure;Figure4.tif

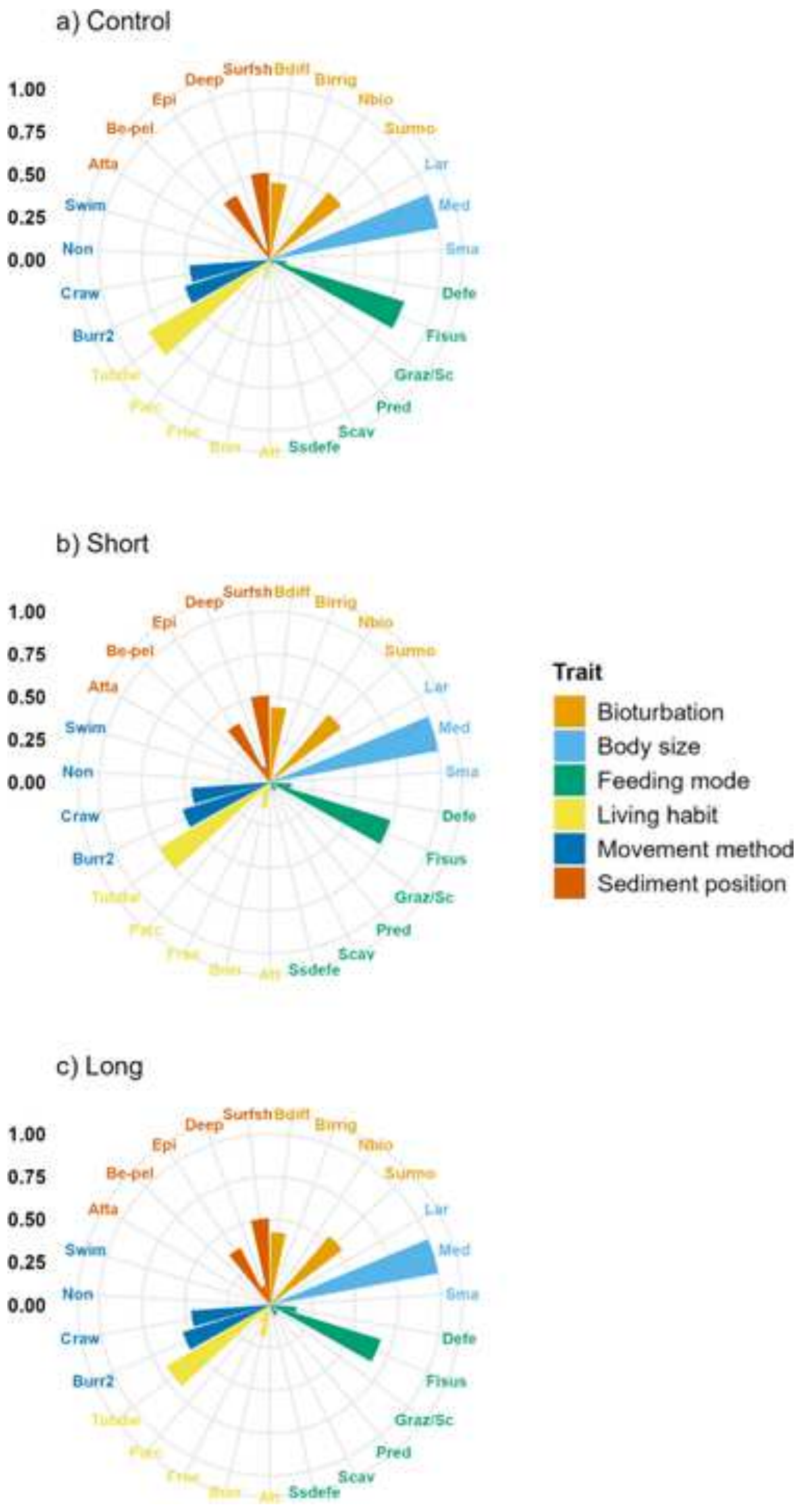

Figure5 Click here to access/download;Figure;Figure5.tif

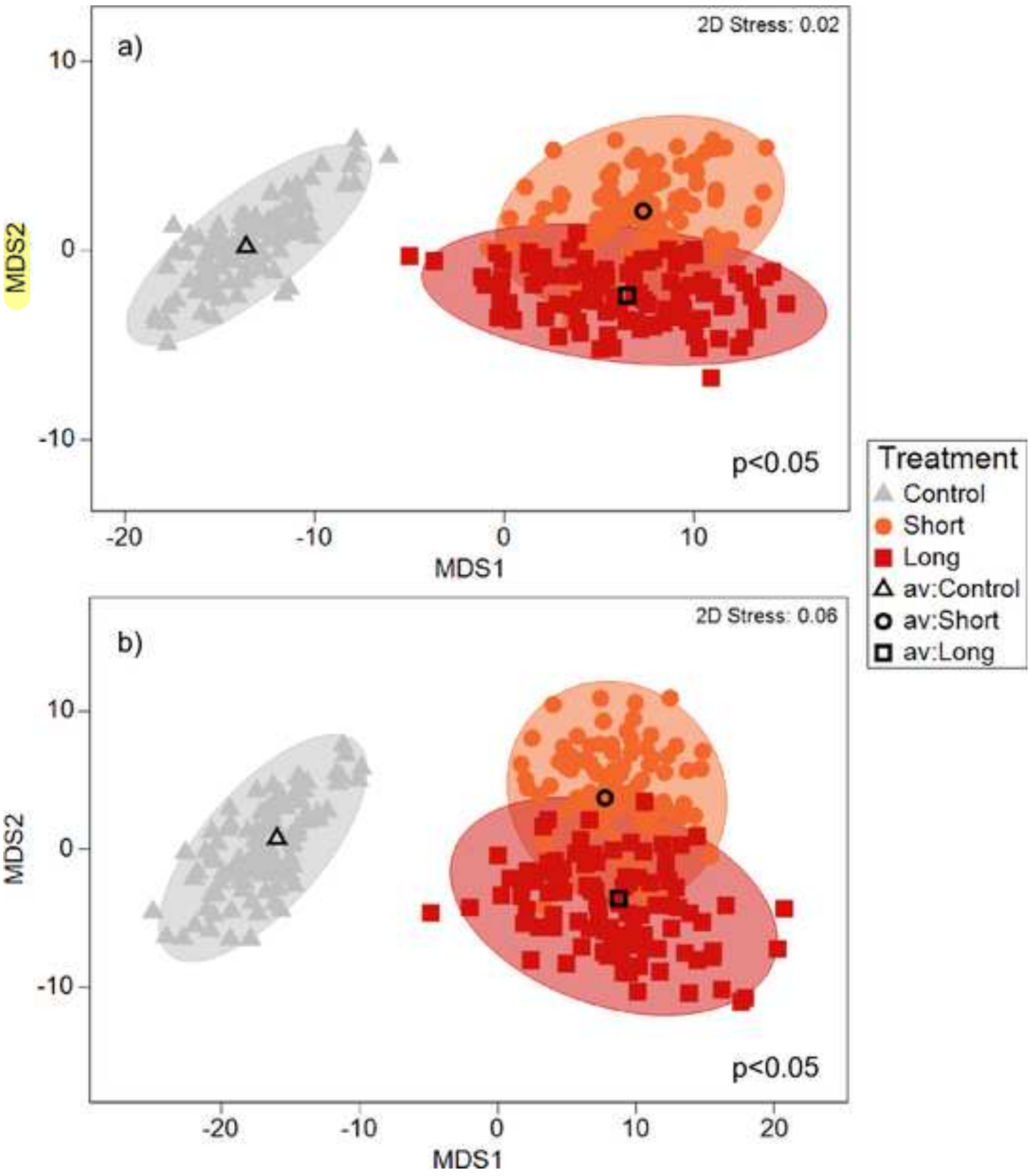

Figure6

Click here to access/download;Figure;Figure6.tif

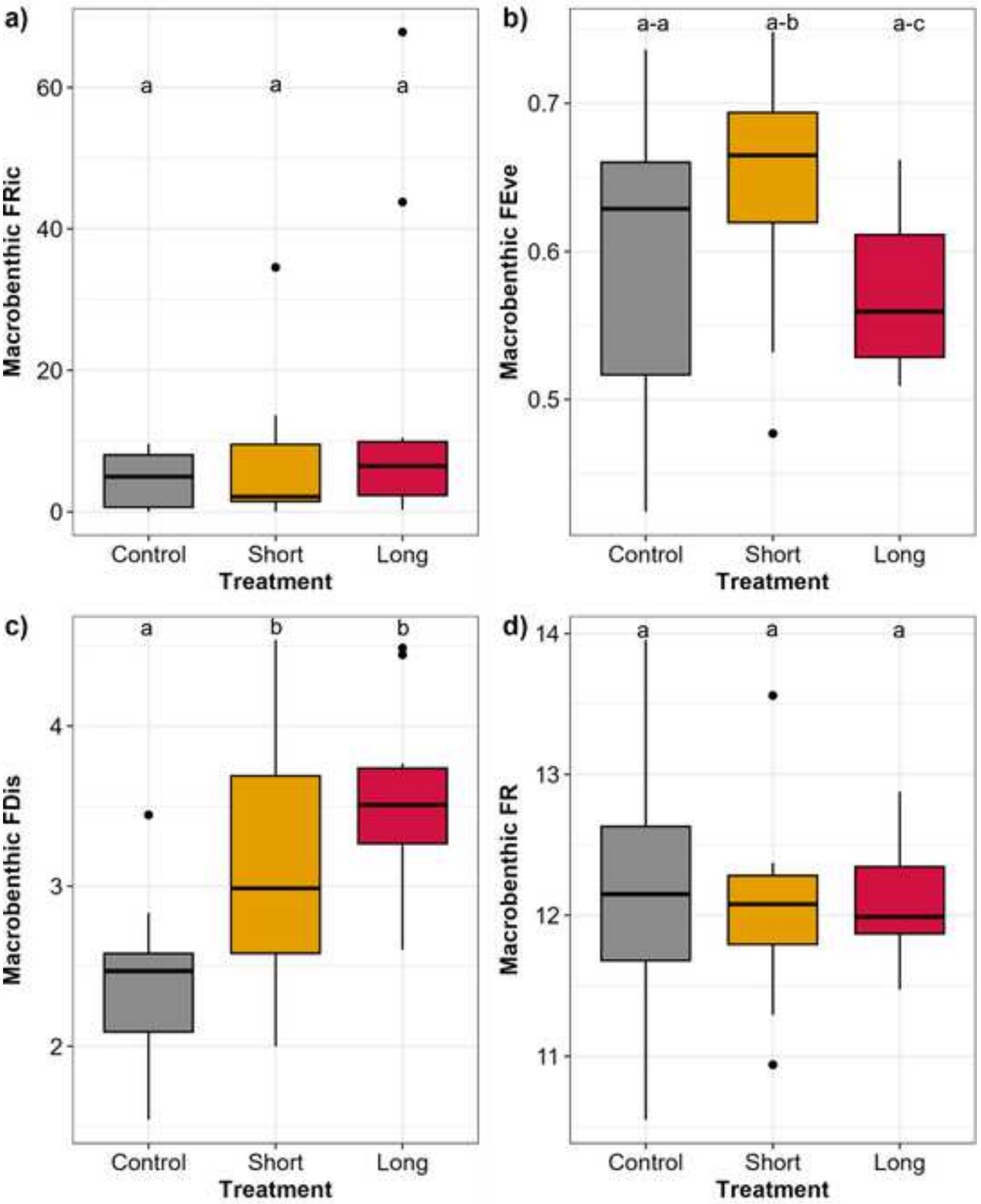

Figure7

Click here to access/download;Figure;Figure7.tif

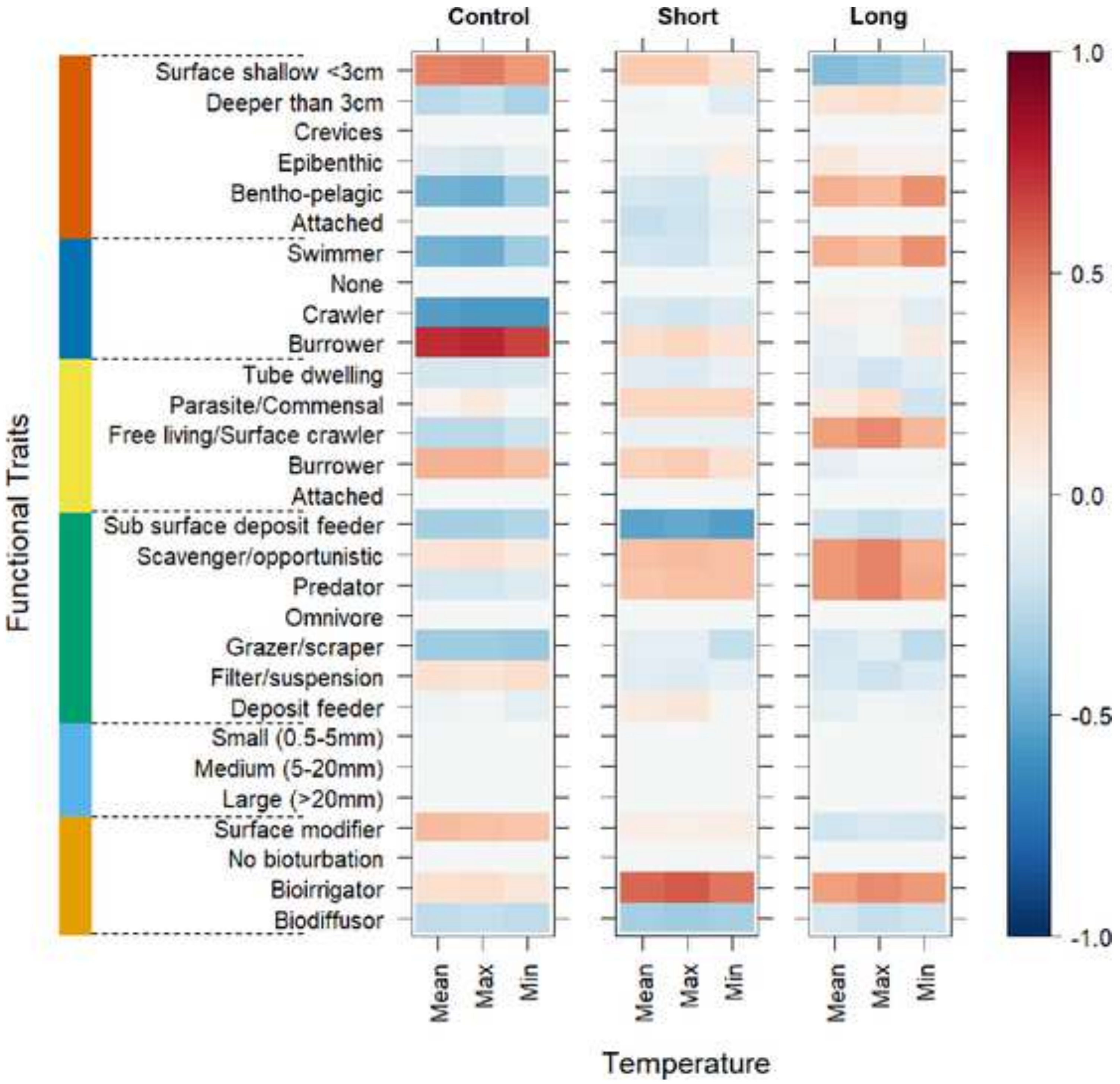

Figure8

Click here to access/download;Figure;Figure8.tif

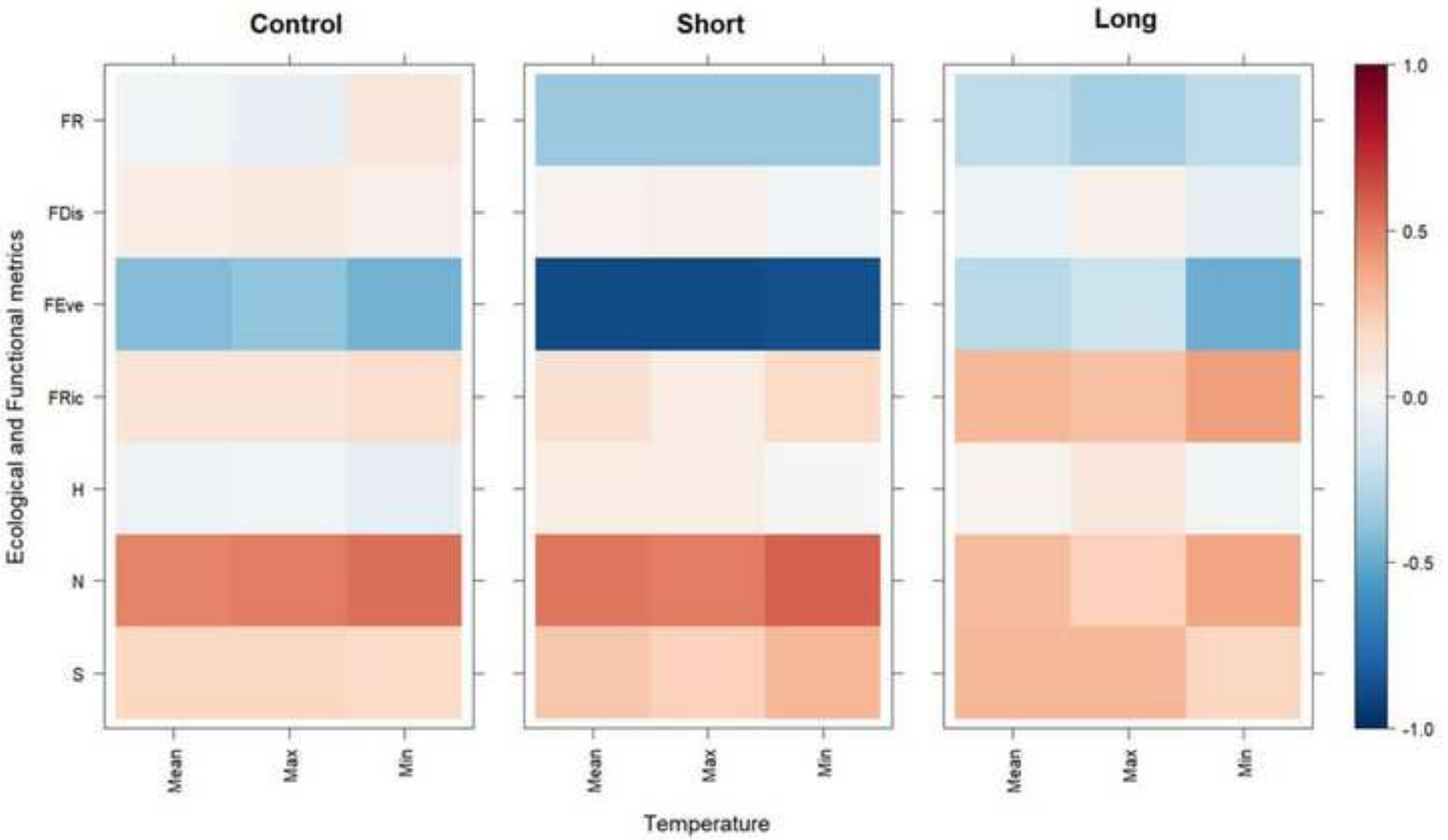

Click here to access/download
Supporting Information
Supplementary materials.docx
