# Supplementary material for: Redundancy of macrobenthic functional traits boosts resilience to a simulated heatwave
Source: PLoS One. 2026 Jan 12;21(1):e0340819. doi: 10.1371/journal.pone.0340819 (PMC12795362; doi:10.1371/journal.pone.0340819)
Supplement: Attachment — Submitted filename: Re-Review assignment for PONE-D-25-44021.pdf [file pone.0340819.s011.pdf]

Re-Review assignment for PONE-D-25-44021: Redundancy of macrobenthic functional traits boosts resilience to a simulated heatwave

I would like to thank the authors for carefully revising this manuscript, which I still believe may be an excellent contribution to the study and application of functional metrics, necessary to understand and predict how ecosystems may respond to the effects of global climate change.

Overall, the authors have thoroughly revised the manuscript by checking and correcting inconsistencies between the main text and the graphs. They have also properly integrated the manuscript with the required information and explanations regarding the experimental design and methodologies used, as requested. Furthermore, they have provided sound and valid counterarguments to the comments expressed in the introduction and discussion sections, and have rightly added supplementary information, allowing for a better elaboration of the data obtained and a clearer interpretation of the results.

In light of the comments and revisions made, I support the acceptance of the manuscript. However, I ask the authors to further check and clarify a few aspects that have emerged after a careful assessment of the data provided in the supplementary materials, which were not previously presented.

I consider this further minor revision a crucial step towards ensuring the scientific validity and consistency of the study, and ultimately necessary for acceptance.

Below, you can find some additional comments concerning the "Data analysis" section and the "Results".

For easier reading, I am also attaching a PDF file containing this information in this format.

Materials and Methods

Data analysis

Line 188 and S1 Table: Firstly, thank you for providing this table. The authors mentioned that no data standardisation or normalisation was performed. Clarification of the values presented in the tables is required. Is this combined matrix obtained by merging species abundances with functional categories? Such merging would provide more reliable quantitative information regarding the functional aspects of the community. Otherwise, this table appears to provide only qualitative information related to the presence of each species or taxon and their relative affinity for certain trait modalities, without weighting by the number of individuals (i.e. abundance) found. Consequently, the values are likely to be low in dispersion, and there may be no need for standardisation or normalisation. In my opinion, a quantitative trait analysis would be more informative in the context of this study, and I would suggest considering this type of analysis, as it could add robustness to the work. I would like to clarify that the procedure performed by the authors is correct, but it may be less informative and contextualised, since variations in species abundances are primarily indicative of changes in community structure when exposed to disturbance. If the authors wish to consider my suggestion to obtain a new merged species-trait matrix weighted by abundance, it is necessary to ensure consistency in the calculation of other functional metrics, including CWM. In this case, standardisation or normalisation may also be required. For example, the high abundance of the amphipod *Paracorophium excavatum* may not be comparable with the lower abundances of other species (e.g. *Paradoneis lyra*).

Lines 205-209: It is stated that "Euclidean distance" was used for both structural metrics (i.e. abundances, S, and H') and functional metrics (FRic, FEve, FDis, FR, and CWM). However, as the

functional trait analysis includes both categorical traits (e.g. feeding mode, movement method, etc.) and continuous traits (e.g. body size), the literature recommends using the Gower dissimilarity index (Gower, 1971; Teichert et al., 2017). Euclidean distance is typically applied only to continuous (environmental) variables.

Results

Lines 299-301: The FRic values are surprisingly high given the number of species found (25). A high FRic indicates a very wide trait space, and this index is usually closely linked to species richness.

Lines 304-305: Please verify that the FEve values exactly match those provided in Table S6. (FEve values range from 0 to 1.)
