# Supplementary material for: Redundancy of macrobenthic functional traits boosts resilience to a simulated heatwave
Source: PLoS One. 2026 Jan 12;21(1):e0340819. doi: 10.1371/journal.pone.0340819 (PMC12795362; doi:10.1371/journal.pone.0340819)
Supplement: Attachment — Submitted filename: Reply_point_by_point_PlosOne_DL_EJD.docx [file pone.0340819.s012.docx]

**Ref.: Ms. No. PONE-D-25-44021**

Resilience of macrobenthic functional traits to simulated heatwave

Dear Academic Editor Marcos Rubal García,

We thank you and the reviewers for constructive comments and the opportunity to amend the manuscript. In our revision, we have addressed all comments and suggestions from both reviewers.

**RESPONSE TO THE EDITORIAL COMMENTS**

1. Please ensure that your manuscript meets PLOS ONE's style requirements, including those for file naming.

**Reply:** Thank you for noting this. In this revised version of the manuscript, we have reviewed the PLOS ONE style requirements and ensured that the manuscript and all associated files now comply with the prescribed formatting guidelines. Track changes was disabled to avoid an overmarked effect across the manuscript.

2. We noticed you have some minor occurrence of overlapping text with the following previous publication(s), which needs to be addressed.

In your revision ensure you cite all your sources (including your own works), and quote or rephrase any duplicated text outside the methods section. Further consideration is dependent on these concerns being addressed.

**Reply:** Thank you for highlighting this concern. We have carefully reviewed the manuscript to identify and address any overlapping text with our previous publications. In the revised version, all duplicated content outside the Methods section has been rephrased or removed, and appropriate citations have been added for all sources, including our own prior work Lam-Gordillo et al. 2025 (Effects of in situ experimental warming on metabolic expression in a soft-sediment bivalve, Scientific Reports) and Douglas et al. 2025 (Simulated heatwave alters intertidal estuary greenhouse gas fluxes, Nature Communications).

3. Thank you for stating the following financial disclosure:

“This research was funded by the New Zealand Government's Strategic Science Investment Fund (SSIF) to the New Zealand Institute for Earth Sciences (ESNZ; FPRS2606).”

At this time, please address the following queries:

a) Please clarify the sources of funding (financial or material support) for your study. List the grants or organizations that supported your study, including funding received from your institution.

b) State what role the funders took in the study. If the funders had no role in your study, please state: “The funders had no role in study design, data collection and analysis, decision to publish, or preparation of the manuscript.”

c) If any authors received a salary from any of your funders, please state which authors and which funders.

d) If you did not receive any funding for this study, please state: “The authors received no specific funding for this work.”

**Reply:** Thank you for reviewing our financial statement. We have modified our financial disclosure to ensure PLOS ONE requirements are met, it reads as follow:

“This research was financially funded by the New Zealand Government's Strategic Science Investment Fund (SSIF) to the New Zealand Institute for Earth Sciences (ESNZ; FPRS2606, FPRS2604). The funders had no role in study design, data collection and analysis, decision to publish, or preparation of the manuscript”

4. Thank you for stating the following in the Funding Section of your manuscript:

“This research was funded by the New Zealand Government's Strategic Science Investment Fund (SSIF) to the New Zealand Institute for Earth Sciences (ESNZ; FPRS2606).”

We note that you have provided funding information that is currently declared in your Funding Statement. However, funding information should not appear in the Acknowledgments section or other areas of your manuscript. We will only publish funding information present in the Funding Statement section of the online submission form.

Please remove any funding-related text from the manuscript and let us know how you would like to update your Funding Statement. Currently, your Funding Statement reads as follows:

“This research was funded by the New Zealand Government's Strategic Science Investment Fund (SSIF) to the New Zealand Institute for Earth Sciences (ESNZ; FPRS2606).”

Please include your amended statements within your cover letter; we will change the online submission form on your behalf.

**Reply:** As suggested by the editor, in this revised version of the manuscript, we have removed the funding statement from the manuscript, and included in this response letter:

“This research was financially funded by the New Zealand Government's Strategic Science Investment Fund (SSIF) to the New Zealand Institute for Earth Sciences (ESNZ; FPRS2606, FPRS2604). The funders had no role in study design, data collection and analysis, decision to publish, or preparation of the manuscript”.

5. We note that Figure 1 in your submission contain [map/satellite] images which may be copyrighted. All PLOS content is published under the Creative Commons Attribution License (CC BY 4.0), which means that the manuscript, images, and Supporting Information files will be freely available online, and any third party is permitted to access, download, copy, distribute, and use these materials in any way, even commercially, with proper attribution. For these reasons, we cannot publish previously copyrighted maps or satellite images created using proprietary data, such as Google software (Google Maps, Street View, and Earth).

**Reply:** Thank you for raising this point. Figure 1 uses imagery sourced from Land Information New Zealand (LINZ), which provides freely available geospatial data under an open license. These images are not proprietary and comply with the Creative Commons Attribution License (CC BY 4.0) requirements for PLOS ONE. We have included proper attribution in the figure caption and confirmed that the imagery is legally permissible for publication. Caption reads: “Fig 1. Map of the study area showing (a) the location of Waihī Estuary in New Zealand, and (b) the area of Waihī Estuary where (c) the experimental plots were located. (d) Close-up view of OTC showing position of stake with temperature loggers attached. Imagery retrieved from Land Information New Zealand (https://data.linz.govt.nz/).

6. Please include captions for your Supporting Information files at the end of your manuscript, and update any in-text citations to match accordingly.

**Reply:** As suggested by the editor, in this revised version of the manuscript we have included the captions for the Supporting information, lines 843-861.

Dear Authors, two external reviewers have now assessed your manuscript "Resilience of macrobenthic functional traits to simulated heatwave”, providing the comments that are reported below. As you can see, they both found your study interesting and generally worth of publication. At the same time, however, they identified a few issues that would require careful revision before this paper is recommendable for acceptance. Note that reviewer 2 had noticed some overlapping of this manuscript with other published and under review papers. Authors must pay special attention to this issue and provide clearly independent results that justify this paper.

**Reply:** Thank you for your feedback and for summarizing the reviewers’ assessments. We appreciate the recognition of the study’s relevance and the opportunity to improve the manuscript. In this revision, we have carefully addressed all reviewer comments and editorial suggestions. Specifically, we have ensured that the manuscript presents clearly independent results and interpretations that distinguish it from our previous related publications. Overlapping content has been minimized by rephrasing, removing redundant sections, and referencing previously published work where appropriate. Additionally, we have emphasized the novel contribution of this study, i.e., the effect of a simulated heatwave on macrobenthic functional traits, which was not covered in the other manuscripts. These changes aim to strengthen the originality and clarity of our manuscript.

Based on the reviewers' and my own assessment, I'm thus here inviting you to take all of these comments into careful consideration and to modify your manuscript according to the provided constructive suggestions. I will then be happy to receive and further examine your revised version together with a point-by-point reply to each comment by myself and each reviewer, where you will need to explain any changes done to a particular piece of text, or include supported and convincing counterarguments to any points you may disagree with I'm confident you will find the present comments and suggestions relevant and useful to improve your work and I'm thus looking forward to hearing back form you by the due time.

**Reply:** Thank you for your guidance and for summarizing the reviewers’ feedback. We appreciate the opportunity to improve the manuscript and have carefully considered all comments and suggestions. In this revision, we have provided a detailed point-by-point response to each comment from both reviewers and the editorial comments, explaining the changes made or providing reasoned clarifications where necessary. Our goal has been to ensure that the revised manuscript fully addresses the concerns raised and meets the standards for clarity, originality, and scientific rigor. We trust that these revisions strengthen the manuscript and look forward to your further assessment. Please see below the detailed reply to the reviewers’ comments.

**RESPONSE TO THE REVIEWER’S COMMENTS**

**REVIEWER #1:**

First, I would like to thank the authors for this interesting work, which I believe can make an excellent contribution to the study of functional diversity and the application of its metrics, which are necessary for a better understanding of how ecosystems function in the context of global climate change. Below is a general comment on the manuscript, followed by comments on each section.

**Reply:** Thank you for your positive assessment of our work and for recognizing its potential contribution to the study of functional diversity and ecosystem functioning under climate change. We appreciate your constructive feedback and have carefully considered all subsequent comments to improve our manuscript. In the revised version, we have addressed each point in detail and provided explanations or clarifications where necessary to ensure the manuscript meets the highest standards.

Overall, the study is well conceptualised and structured, with a solid foundation in marine community ecology. The experimental concept is promising and provides valuable and interesting insights into how marine macrofaunal communities respond to the main effects of climate change, to which they are increasingly exposed. The experimental design is well constructed and balanced, supported by an adequate number of replicates, which gives the study good statistical representativeness and robustness.

However, in reporting and describing the study, several gaps were identified, particularly regarding sample collection, and an adequate description of the data processing for the calculation of the functional metrics, which are central to the study, was not provided.

**Reply:** Thank you for your positive evaluation of the study’s conceptualization and design. We appreciate your recognition of the robustness of the experimental approach and its relevance to understanding climate change impacts on marine macrofaunal communities. Your feedback reinforces the importance of this work, and we have taken care to maintain the clarity and rigor of the manuscript while addressing all subsequent comments to further strengthen its contribution.

In the revised manuscript, we have added detailed descriptions of the sample collection procedures and clarified the steps involved in data processing for the calculation of functional metrics in the Methods section. Specifically, we have now explained how the species abundance matrix and trait matrix were prepared, including fuzzy coding, standardization, and the methods used to compute single-trait and multi-trait indices, in addition of presented the macrobenthic abundance raw data in the supplementary materials. These clarifications aim to improve transparency and allow readers to fully understand the analytical workflow.

As a result, it was not possible to consult the data (including means and standard deviations) obtained, as these were not clearly presented in either the main text or the supplementary materials. This makes it difficult to interpret the results, which are displayed only in the graphs, and also hinders comparison with other studies and a thorough evaluation of the work carried out.

**Reply:** Thank you for highlighting this issue. In the revised manuscript, we have included the macrobenthic abundance raw data in the supplementary materials as a new table to ensure transparency and facilitate comparison with other studies. Additionally, we have referenced these values in the main text where relevant to support interpretation of the graphical results. These additions aim to improve clarity and allow for a more thorough evaluation of the work.

For easier reading, I am also attaching a PDF file containing this information in this format, as well as a PDF file of the manuscript with the corresponding comments.

INTRODUCTION
Overall, the Introduction is well written, and the objectives of the study are clearly explained. However, it requires some additions. As the study mainly focuses on the “functional” aspects of the infaunal community, a brief, in-depth description and definition of functional diversity and its metrics is needed.

**Reply:** Thank you for the positive comments. As suggested by the reviewer we have provided the definition of functional diversity and related metrics in the introduction section. Lines 82, and 88-96.

78: “Functional diversity also plays a critical role in resilience.” This is true, but it would be useful to spend some time explaining what functional diversity is and how it can be measured. I suggest briefly describing the metrics used to estimate functional diversity (which is not straightforward) and providing some references. Describing these metrics may help readers, including non-specialists, to better understand your results.

**Reply:** As suggested by the reviewer, in this revised version of the manuscript we have provided the definition of functional diversity and further explain how it is calculated. We also have detailed the metrics related to functional diversity and provide the relevant references in the Introduction section, lines 88-96, and provided extended clarification on the calculations and metrics used in the Methods section, lines 206-208.

94-95: “functional metrics (i.e., individual functional traits, functional indices)”. It is not clear what type of indices you include under the definition of “individual functional traits”. In particular, it is not clear from the initial reading whether this refers to the organism level or to a single functional trait. I assume it refers to a single functional trait. If so, I suggest changing “individual functional trait” to “single-trait indices” and “functional indices” to “multi-trait indices”. “Single-trait indices” include, for example, the CWM that you mention later, while “multi-trait indices” include “functional richness, evenness”, etc. It would be more accurate to refer to these broader categories.

**Reply:** As suggested by the reviewer, in this revised version of the manuscript we have modified the wording and adopted the terms “single-trait indices and multi-trait indices”. Lines 114-116, “… and functional metrics (i.e., single-trait indices (Community-weighted means), and multi-trait indices (Functional Richness, Evenness, Dispersion, Redundancy))…”.

MATERIALS AND METHODS
The study and its objectives are promising, and it may provide new perspectives on understanding the responses of macrobenthic infaunal communities to more frequent events driven by global climate change. However, some aspects need clarification, from the experimental design and sample collection to data processing and analysis. Furthermore, for greater accuracy, it would be useful to include separate tables with the abundance matrix and species-trait matrix, perhaps in the supplementary materials. This would allow for a better understanding of the results.

**Reply:** Thank you for your positive assessment of the study and for highlighting areas that require clarification. In the revised manuscript, we have expanded the descriptions of the experimental design, sample collection procedures, and data processing steps to ensure transparency and reproducibility. Additionally, we have included separate tables for the species abundance matrix (S2 Table) and the species–trait matrix (S3 Table) in the supplementary materials, as suggested.

Figure 1. Please check the caption and ensure that the letters correspond accurately to those indicated in the figure. Correct "e" to "d" in the figure.

**Reply:** As suggested by the reviewer, we have corrected the caption of Figure 1.

111: Since you are discussing the OTCs, please refer directly to Figure 1d.

**Reply:** As suggested by the reviewer, we have directly referred to Figure 1d.

117-118: It is not clear whether the OTCs were removed during the night and at high tides. Please clarify.

**Reply:** A suggested by the reviewer, in this revised version of the manuscript we have clarified that the OTCs were removed from the experimental plots during the night and at high tides. Line 149.

119: For greater clarity, please specify that the “Control treatment” is without the OTCs.

**Reply:** As suggested by the reviewer, we have clarified that the Control treatment was without OTCs. Line 151.

129: Did you remove the temperature loggers from the control plots during the night and at high tide? How long is the tidal excursion? Did the logger measure temperature only in the top 1 cm of the sediment? If so, how can you be sure that the temperature also increases in the lower layers?

**Reply:** Thank you for raising these important points. In the revised manuscript, we have clarified the handling of temperature loggers and sediment temperature measurements. The loggers were not removed during the night or high tide; they remained in place throughout the experiment to ensure continuous temperature recording. The tidal range in the study area is approximately 2.5–3 meters and the site was exposed to air for approximately 5-6 hours during each tidal cycle (spanning the predicted low tide time but also depending on atmospheric conditions and wind strength/direction). The loggers recorded temperature at the sediment surface (0–1 cm), which represents the most thermally responsive layer during low-tide exposure. While deeper layers were not directly measured, previous studies and thermal diffusion models indicate that heat transfer occurs downward, albeit attenuated, during prolonged exposure. We have added this clarification to the Methods section, lines 132 and 168-169.

132-137: You mentioned the use of a PVC corer for macrofaunal sampling. How many cores did you collect per plot? Please specify. Furthermore, you used a PVC corer with a depth of 15 cm for macrofaunal sampling. Based on this, did you observe any sediment stratification? Did you consider all the macrofauna within the 15 cm, or did you collect it only from the surface layers? As you correctly mentioned in the introduction, some species may respond to thermal increases by moving within the sediment. Therefore, it would be interesting to observe this in the cores. Additionally, the procedure for macrofaunal sampling is not very clear. Please clarify: once collected, was the core rinsed and sieved through a 0.5 mm mesh?

**Reply:** Thank you for asking these important questions. In the revised manuscript, we have clarified the macrofaunal sampling procedure in the Methods section. Specifically, we collected one core per plot using PVC corers (15 cm depth), and all macrofauna within the full 15 cm depth were considered. While we did not observe distinct sediment stratification during sampling, the entire core was processed to capture potential vertical movements of organisms in response to thermal stress. Immediately after collection, each core was sieved through a 0.5 mm mesh to retain macrofaunal specimens. These details have been added in lines 166, and 171-172.

160: Regarding Table 1, there is an inconsistency between the acronyms and the trait modalities for the traits “Living Habit”, “Movement Method”, and “Sediment Position”. Please check and correct.

**Reply:** Thank you for pointing this out. We have carefully reviewed Table 1 and corrected the inconsistencies between the acronyms and the trait modalities for “Living Habit,” “Movement Method,” and “Sediment Position.”

165-169: It is not clear how you calculated the four metrics, particularly which data you used. Generally, obtaining these metrics requires a combination of the species abundance matrix and the trait matrix (with fuzzy coding). For greater clarity, please revise this section to specify how you treated the data before calculating the metrics. This clarification would help readers understand the results obtained. If you used a different procedure, please specify it. Also, indicate whether you performed standardisation or transformation of the data.

**Reply:** Thank you for highlighting this point. In the revised manuscript, we have clarified the procedure for calculating the four functional diversity metrics in the Methods section. These metrics were derived using a combination of the species abundance matrix and the species–trait matrix, with traits assigned through fuzzy coding and using the R package FD. We did not standardize or normalize the data as there was no overdispersion in the raw data. Lines 206-208.

174: Are the factors mentioned related to the simulated heatwaves (5 days and 7 days)? This is not clear. Please specify the factors considered and the levels for each factor.

**Reply:** In the revised manuscript, we have clarified the factor “treatment” considered in the statistical analyses. The factor was treatment, with three levels: Control (no OTC), Short (5-day exposure), and Long (7-day exposure). Line 213.

172-178: This section is somewhat difficult to follow and may cause confusion. PERMANOVA was used to test the effects of MHWs on taxonomic and functional metrics, correct? If so, it is redundant to restate the factors. If not, it is unclear which factors are being tested. As mentioned above, please specify the factors being analysed. Additionally, the phrase “the Euclidean distance for the single macrobenthic metrics” is unclear; it is not specified which metrics are being referred to. I recommend clarifying this short paragraph to avoid misunderstandings regarding the statistical analysis performed.

**Reply:** As suggested by the reviewer we have clarified this paragraph, also taking into account the previous comment. Lines 213-218.

183: Do “Functional trait data” refer to the combination of the macrofaunal data and trait “fuzzy coding assignation”?

**Reply:** Yes, it refers to the combination of macrobenthic abundance data and macrobenthic functional trait fuzzy coding data. In this revised version of the manuscript, we have added this clarification. Lines 223-224.

184-187: This section is clearer than the previous one. I suggest making changes to the previous section, using this one as a starting point.

**Reply:** As suggested by the reviewer, we have made the relevant changes to make the section clearer. Lines 213-218.

RESULTS
Overall, the results are well presented and consistent with the Materials and methods section. However, I suggest further integration and more in-depth descriptions in the main text, as well as modifications to the plots. Generally, the choice of plot types is good, and they are easy to read, even for non-specialists. However, some plots need to be checked and adjusted to ensure consistency with the descriptions in the main text.

**Reply:** Thank you for your positive assessment of the results presentation and for suggesting improvements. In the revised manuscript, we have integrated more detailed descriptions into the main text to strengthen the interpretation of the findings. Additionally, we have reviewed and adjusted the plots to ensure consistency with the text and improve clarity.

214-228: I suggest providing a supplementary table with the abundance and diversity data to support the interpretation of the boxplots. It may also be useful to describe in the main text the magnitude of these changes among the treatments (even if not significant in some cases), including values, as has already been done previously for temperature.

**Reply:** Thank you for this suggestion. In the revised manuscript, we have added 5 new supplementary tables that include the abundance and functional trait data (Supplementary tables S2, S3, S4, S5, and S6). Additionally, we have incorporated descriptions in the main text to indicate the magnitude of changes among treatments, including numerical values where relevant, even when differences were not statistically significant. Lines 259-272.

236-237: Please specify the p value.

**Reply:** A suggested by the reviewer, we have included the p-value, “p<0.05”. Line 286. Detailed p-values are presented in Table 2.

247-252: How does the community structure change among the treatments? I suggest providing a description of the community structure in all treatments and indicating which species or taxa drive the arrangement in the nMDS. The same applies to the functional trait composition. It is also possible to overlay as vectors the species that contribute most to these dissimilarities. Please take this into consideration. Otherwise, it must be specified which main differences are supported by the statistical analysis.

**Reply:** Thank you for this suggestion. We performed a bootstrapped non-metric multidimensional scaling (nMDS) analysis, which does not allow for overlaying species or trait vectors. To address your concern, we have now provided a detailed description of the community structure and functional trait composition in the main text, highlighting the taxa and traits that contributed most to the observed differences supported by the statistical analysis. Lines 299-301 and 304-307.

254–265: As in the previous comment, for greater comprehensiveness, it may be useful to provide at least the mean values and standard deviations of the multi-trait indices (FRic, FEve, etc.) to better understand the magnitude of the differences. I suggest adding a supplementary table with the means of all the descriptors considered (abundance, richness, diversity, FRic, FEve, etc.) to support the reading of the main text and the plots.

**Reply:** Thank you for this suggestion. In the revised manuscript, we have added a supplementary table (table s5) that includes the mean, max, and min values for all the relevant metrics (FRic, FEve, FDis, FR) to support interpretation of the boxplots. Additionally, we have incorporated these values into the main text where relevant to provide context. Lines 309-326.

270-272: Figure 7. In the caption, the sentence “The colour scale indicates the magnitude of the correlations” is assumed to refer to the colour gradient bar on the right. Does it represent the interval of the Spearman correlation coefficient (rho (ρ) = +1, 0, -1)? If so, please specify this in the caption and also next to the scale bar. Furthermore, if this is the case, there appears to be an inconsistency between the main text and Figure 7. According to the main text, a significant negative Spearman correlation was found between the Control and the trait modality “burrower”. In Figure 7, a negative correlation would be expected to be shown with a “blue” shade. Is the colour scale inverted? Please clarify this aspect to avoid misinterpretation of the results.

**Reply:** Thank you for noting this issue. You are correct that the color scale in Figure 7 was inverted in the original version. We have corrected this inconsistency in the revised manuscript by updating the figure caption to specify that the color gradient represents the Spearman correlation coefficient (ρ), ranging from +1 (strong positive correlation, red color) to −1 (strong negative correlation, blue color), and by ensuring that the interpretation in the main text aligns with the corrected scale.

277-283: Figure 8. There is the same issue with the visualisation of the correlation. It appears that the interval is inverted: positive correlations are blue and negative correlations are red. Please ensure that the interval on the right matches exactly with the output of the level plot, as it currently appears inconsistent. As previously suggested, if the interval on the right (+1 / -1) corresponds to the Spearman coefficient (rho), please specify this next to the gradation scale in the plot and/or in the figure caption.

**Reply:** As suggested previously by the reviewer, we have made the relevant changes in figure caption and text in results section.

DISCUSSION
This section provides an overview of the study and its findings. However, although the arguments are valid and appropriate, they seem to enhance and reinforce the study and its findings only partially. The study required significant practical and interpretative effort, which I believe deserves greater recognition. While it is acknowledged that interpreting functional diversity metrics is not easy, if these metrics are used, it is necessary to provide a valid explanation of their selection and the type of information they convey. Only some of these metrics are explained, while others, which are also informative, are omitted. Furthermore, the arguments supporting ‘functional’ resilience are unconvincing because they rely solely on evidence of redundancy, which may result from bias in the selection of functional traits. I recommend that the authors supplement their argument by providing supporting data, for example in the form of tables. This would lend further weight to the arguments presented in this section and give due recognition to the work undertaken.

**Reply:** Thank you for this valuable feedback. In the revised manuscript, we have expanded the Discussion section to provide a more comprehensive explanation of the functional diversity metrics used, including their ecological relevance and the type of information they convey. We have also addressed metrics that were previously omitted and clarified their interpretation in the context of our findings. To strengthen the arguments supporting functional resilience, we have supplemented the discussion with additional supporting data presented in the new tables in the supplementary materials. We have made changes throughout the Discussion section.

293-294: In my opinion, the findings related to the functional traits and metrics do not necessarily indicate resilience of the community but rather demonstrate redundancy in functional diversity during the experiment. Therefore, the sentence “however, functional traits and functional metrics were less affected, suggesting a degree of functional resilience” is problematic. Firstly, since Functional Evenness and Functional Dispersion changed significantly according to your results, this is inconsistent with your statement. Secondly, as I understand it, the experiment simulates a heatwave during low tide with relatively short exposure (4 hours per day, every day), alternating with high tide. Is this correct? Do you think this exposure is sufficient to expect changes in functional traits? Additionally, providing information on how many cores were collected from each plot each day would help to support this statement. Furthermore, a brief description of the community in the “Results” section could also help to predict the response to the heatwave experiment.
Furthermore, in selecting the functional trait, I notice some overlap between Living Habit and Movement Method, for example in the modalities “Crawler” and “Burrower”. The choice of trait depends on the structure of the community, and sometimes different species may share the same functional trait. Since trait selection is arbitrary and there is no standard rule for how many or which traits to consider, have you tried considering only one of the two functional traits in question—either “Movement Method” or “Living Habit”? This might reduce overlap and redundancy. How can you explain that the observed redundancy is a response of the community to the HWs, rather than a redundancy resulting from the “over-expression” of the same trait due to overlap between trait modalities?

**Reply:** Thank you for raising these important points. We acknowledge that interpreting functional resilience requires careful consideration of multiple metrics and trait selection. In the revised manuscript, we have addressed these concerns as follows:

(1) Clarification of resilience vs. redundancy: We have revised the statement to avoid conflating resilience with redundancy. The discussion now emphasizes that observed functional redundancy may buffer ecosystem functioning against short-term disturbances, but does not necessarily imply full resilience. This distinction is clearly articulated in the revised text. Lines 355-356.

(2) Significant changes in FEve and FDis: We have incorporated an explanation of the observed changes in Functional Evenness and Functional Dispersion and discussed their ecological implications, rather than presenting them as evidence of resilience. Lines 404-416.

(3) Exposure duration and experimental design: Yes, the experiment simulated heatwave conditions during low tide for approximately 4 hours per day over 5- and 7-day periods, alternating with high tide. We have clarified this in the Methods section and acknowledged in the Discussion that this exposure represents short-term stress, which may limit the extent of functional trait shifts. Lines 457-467.

(4) Sampling details: We have added information on the number of cores collected per plot (one per plot) and included a brief description of the community structure in the Results section to provide context, as previously suggested by the reviewer.

(5) Trait overlap and redundancy: We recognize the potential nominal overlap between “Living Habit” and “Movement Method”, however this overlap is only nominal, as the behavior of the organisms in terms of living habit and movement method could vary. In the revised manuscript, we have explained the rationale for including both traits, as they capture different ecological dimensions despite some shared modalities. The rationale about the selection of functional traits is presented in methods section. Lines 197-199.

(6) Supporting data: To strengthen our argument, we have added 4 new supplementary tables with detailed macrobenthic metric and trait values.

296–298: Does the increase in temperature refer only to the first centimetre (0–1 cm) of the sediment? Did you observe thermal increases below the first sediment layer as well? If so, as you correctly mentioned in the introduction, some species might move within the sediment. In this case, documenting the effect of temperature increase could be challenging if the data refer only to the sediment surface.

**Reply:** Thank you for raising this point. In the revised manuscript, we have clarified that temperature measurements were taken at the sediment surface (0–1 cm), which represents the most thermally responsive layer during low-tide exposure. We did not directly measure temperature below this layer; but we are now considering it in future research. We have acknowledged this limitation in the Discussion and explained that surface measurements are ecologically relevant because many macrobenthic species occupy or interact with the upper sediment layer during low tide. Lines 383-388.

330-346: What about the other indices, Functional Evenness and Functional Dispersion? According to your results, they change significantly between the control and treatments, as shown in the boxplot in Figure 6. It would be noteworthy to comment on their trends. Why does FDis increase significantly with exposure to the HW? The same question applies to FEve.

**Reply:** Thank you for highlighting this point. In the revised manuscript, we have added a discussion of the trends observed for Functional Evenness (FEve) and Functional Dispersion (FDis). Lines 404-416.

**REVIEWER #2:**

The authors conducted an in-situ warming experiment using open-top chambers (OTCs) in an intertidal estuary to assess heatwave-induced changes in macrofaunal community composition and functional trait resilience. The benthic community was sampled after 5 and 7 days of heating to additionally test the effect of increased warming duration. A key finding of this study is the persistence of community functions due to functional redundancy after a change in macrofaunal abundance and diversity.

The topic and overall objectives of this study are timely and relevant, and the results have the potential to contribute valuable insights into the resilience of intertidal benthic communities to heatwaves. However, the manuscript in its current form requires significant revision before it would be suitable for publication in a high-impact journal.

**Reply:** Thank you for your positive assessment of the study’s relevance and objectives. We appreciate your recognition of its potential contribution to understanding the resilience of intertidal benthic communities under heatwave conditions. In the revised manuscript, we have carefully addressed all reviewer comments and made substantial improvements.

To my knowledge, this is the third manuscript arising from the same experimental setup, alongside the already published Lam-Gordillo et al., 2025 (“Effects of in situ experimental warming on metabolic expression in a soft-sediment bivalve”) and Douglas et al., in review (“Simulated heatwave alters intertidal estuary greenhouse gas fluxes: duration and degradation state determine response”). Given this context, it is essential that the present manuscript clearly articulates what new knowledge is being provided beyond those studies.

At present, there is substantial overlap in both methodology and findings. For example, the section “Simulated marine heatwave: intertidal sediment warming” closely replicates previously published results (e.g., “Seafloor temperature and sediment characteristics” in Lam-Gordillo et al., 2025. Additionally, a large part of the community composition results reiterates material already addressed in Douglas et al., in review. This leaves the functional-trait component as the primary novel contribution; however, as these analyses are limited to a single experimental site, the study does not fully leverage the broader context (muddy vs. sandy sites) that has been central to the complementary papers.

To strengthen the manuscript’s contribution, I strongly encourage the authors to (1) streamline or reference previously published overlapping results rather than repeating them, (2) clearly define the unique research question and interpretive framework that distinguishes this study from the others, and (3) consider expanding the functional-trait approach to include data from both experimental habitats where possible, as this would better reveal context-dependent responses and ecological relevance.

**Reply:** Thank you for highlighting this important point and for recognizing the need to clearly differentiate the present manuscript from related studies. We have taken the following steps to address your concerns:

(1) Clarified Novel Contribution: The revised manuscript explicitly states that its primary focus is on the analysis of functional traits and functional metrics to assess redundancy/resilience of macrobenthic functional traits under simulated heatwave conditions, which was not addressed in Lam-Gordillo et al. (2025) or Douglas et al. (2025). This distinction is now emphasized in the Methods sections where we added new text, lines 121-126.

(2) Define the unique research question: We have clarified the particular aims and hypothesis of this study in Introduction section lines 105-118.

(3) Streamlined overlapping content: We have taken steps to minimize overlapping content by summarizing previously published material, referencing relevant sections, and incorporating new analyses where appropriate. However, we retained certain contextual results (while ensuring they are not identical to those in our earlier publications) so that this manuscript can stand alone and be fully understood without requiring readers to consult the complementary studies. This approach maintains clarity and completeness while emphasizing the novel aspects of the present work.

(4) Expanded interpretive framework: We have strengthened the discussion of functional redundancy and resilience, integrating additional ecological context and supporting data from 4 new supplementary tables. This ensures that the manuscript provides a distinct perspective on how short-term heatwaves influence functional diversity metrics.

(5) Acknowledged experimental and study Scope: We have explicitly clarified the rationale for focusing on a single site. The scope and objectives of this study were designed from the outset to examine functional trait responses within one estuarine habitat, allowing for a controlled assessment of short-term heatwave effects without introducing variability from multiple sediment types. This focused approach is a defining characteristic that distinguishes the present work from the complementary studies, which addressed broader habitat comparisons and other ecological processes. We have highlighted this point in the Methods section, lines 133-137.

Language and format editing should also be revised throughout the manuscript.

**Reply:** As suggested by the reviewer, in the revised manuscript we have carefully reviewed and improved the language and formatting throughout to ensure clarity, consistency, and adherence to journal standards. This includes refining sentence structure for readability, correcting minor grammatical issues, and standardizing terminology and style across all sections. We have also verified compliance with PLOS ONE formatting guidelines.

More specific comments below:

Title: Adjust the title to the scope of the study.

**Reply:** As suggested by the reviewer, we have amended the title to better represent our study: “Redundancy of macrobenthic functional traits boosts resilience to a simulated heatwave”

Introduction:

Paragraph 61-65: The previous paragraph states that intertidal macrobenthic communities are generally well adapted to extreme conditions, while this paragraph describes them as highly sensitive. Please clarify these contrasting statements.

**Reply:** Thank you for pointing out this apparent inconsistency. We have revised the text to clarify that while intertidal macrobenthic communities are generally adapted to natural fluctuations such as tidal cycles and seasonal changes, they remain highly sensitive to extreme and prolonged stressors beyond their usual environmental range, such as marine heatwaves. These events can exceed physiological thresholds and disrupt ecological processes despite the communities’ baseline tolerance to variability. The revised paragraph now emphasizes this distinction between adaptation to regular extremes and vulnerability to unprecedented or prolonged disturbances. Lines 58-60 and 67-74.

Methods:

Line 125: “Environmental data” is a bit too general for just including temperature here.

**Reply:** As suggested by the reviewer, we have amended the subheading and changed to “Temperature Data”.

Line 133: The methods mention that there are 10 plots for Control, Short, and Long treatments. Does this mean the Control plots were sampled only after 7 days? If so, could you clarify how comparable a 7-day Control is to the 5-day heating treatment, particularly given the changes in weather conditions during the final two days (Figure 2)? This has important implications for interpretation, as heating and exposure time are confounded and cannot be disentangled.

**Reply:** Thank you for raising this important point. We confirm that treatments Control and Long (7-day exposure) were sampled at the same time after the 7-day experimental period. Short (5-day exposure) treatment was sampled after the 5-day experiment period. The Control plots were not exposed to OTCs at any point; they remained under ambient conditions throughout. We acknowledge that this design means the Short treatment experienced heating for 5 days, while the Control and Long treatments were exposed to natural conditions for 7 days, which introduces a temporal difference. However, it is very unlikely that macrobenthic fauna in the untreated Control plots changed substantially during the additional two days, as these plots were under stable ambient conditions without any imposed stressor. Previous studies and baseline monitoring in similar estuarine habitats indicate that short-term natural variability over two days does not significantly alter macrobenthic community composition or functional traits compared to experimental heating effects (e.g., Gruner et al., 2017; Patrício et al., 2009; Ritter et al., 2005; Ysebaert and Herman, 2002).

To address this, we have clarified in the Methods section that the factor tested was “treatment” (Control, Short, Long), and interpretations focus on heating effects rather than absolute exposure time. We have also added a note in the Discussion acknowledging this limitation and its implications for interpretation. Lines 457-467.

Results:

A detailed Section on “Simulated marine heatwave: intertidal sediment warming” is mostly unnecessary as Lam-Gordillo et al., 2025 already stated that the temperatures are significantly different between OTC and control. Furthermore, Douglas et al. (in review) confirmed significant difference and additionally specifies the actual temperatures. However, a shortened version for the context should remain. But these shortened information can also be presented in the methods section.

**Reply:** Thank you for this suggestion. We acknowledge that some temperature-related results were previously reported in Lam-Gordillo et al. (2025) and Douglas et al. (2025). However, we have decided to retain the detailed section on “Simulated marine heatwave: intertidal sediment warming” because this manuscript is intended to stand alone and provide full context for readers without requiring consultation of complementary studies, as the general pattern is similar, but the presentation of results and fine details are different.

Throughout the results section: Please ensure accuracy when describing the boxplot figures. Boxplots display the median (not the mean), along with the interquartile range and potential outliers. Some statements currently interpret boxplots as showing mean values, which should be corrected for clarity and statistical accuracy (Lines 216-224, 256-260).

**Reply:** Thank you for this comment. As suggested by the reviewer, we have reviewed all descriptions of boxplots in the Results section to ensure statistical accuracy. We have corrected statements that previously referred to boxplots as showing mean values and now explicitly state that they represent the median. These changes have been applied across the whole Results section. In addition, we added 2 new supplementary tables that include the mean, max, and min values to improve clarity and consistency of the results.

Line 238: The statement that “suspension feeders were significantly higher in the control treatment compared to the short and long treatment” seems difficult to evaluate as no quantitative data for suspension feeders are provided. Table 2 only reports test results, and Figure 4 shows proportional feeding mode contributions. Please include the underlying abundance data to support this conclusion.

**Reply:** As suggested by the reviewer, in the revised manuscript we have included the underlying quantitative CWM trait data for all trait modalities in the supplementary materials (S5 Table) to support the interpretation of trait modalities differences among treatments. In addition, we also added relevant values in the Discussion section, lines 395-396.

Line 246: Please clarify if a nMDS or MDS was conducted.

**Reply:** As suggested by the reviewer, we have clarified that we used nMDS plots. This was presented in line 221 in Methods section and in Figure 5.

Lines 256-265: This section exemplifies a need for caution when interpreting statistical comparisons among Control, Short, and Long treatments. As noted above, sampling time and heating duration are confounded, and therefore not all treatment contrasts represent clean tests of heating effects. Please clarify which comparisons are appropriate and adjust interpretations accordingly.

**Reply:** Thank you for raising this important point. We confirm that all treatments, including Control, Short (5-day exposure), and Long (7-day exposure), were sampled at the same time after the 7-day experimental period. The Control plots were not exposed to OTCs at any point; they remained under ambient conditions throughout. We acknowledge that this design means the Short treatment experienced heating for 5 days, while the Control and Long treatments were exposed to natural conditions for 7 days, which introduces a temporal difference. However, it is very unlikely that macrobenthic fauna in the untreated Control plots changed substantially during the additional two days, as these plots were under stable ambient conditions without any imposed stressor. Previous studies and baseline monitoring in similar estuarine habitats indicate that short-term natural variability over two days does not significantly alter macrobenthic community composition or functional traits compared to experimental heating effects (e.g., Gruner et al., 2017; Patrício et al., 2009; Ritter et al., 2005; Ysebaert and Herman, 2002).

To address this, we have clarified in the Methods section that the factor tested was “treatment” (Control, Short, Long), and interpretations focus on heating effects rather than absolute exposure time. We have also added a note in the Discussion acknowledging this limitation and its implications for interpretation. Lines 457-467.

Lines 267-283: Positive and negative correlations described here do not match the colour scale presented in Figure 7. The colour scale shows dark red to be highly positively correlated and dark blue for highly negative correlation. Please clarify and realign your interpretations (or correct the figure).

**Reply:** Thank you for noting this issue. Reviewer 1 also highlight this issue. You are correct that the color scale in Figure 7 was inverted in the original version. We have corrected this inconsistency in the revised manuscript by updating the figure caption to specify that the color gradient represents the Spearman correlation coefficient (ρ), ranging from +1 (strong positive correlation, red color) to −1 (strong negative correlation, blue color), and by ensuring that the interpretation in the main text aligns with the corrected scale.

Discussion:

Line 303-305: This is the first time where actual abundance data are presented. Given that community metrics are a central aspect of the study and can help explain several of the observed responses, it would be important to present these data more comprehensively elsewhere. In addition, when reporting abundance, please provide an appropriate unit (e.g., individuals per core, per square meter). Yet again the question – How reasonable is it to compare the Control abundance to Short treatment abundance?

**Reply:** Thank you for highlighting this important point. This was also highlighted by Reviewer 1. In the revised manuscript, we have presented macrobenthic abundance data more comprehensively by including new supplementary tables (S2-S6 Table) that reports mean, minimum, and maximum values for all descriptors, including macrobenthic abundance, richness, diversity, and functional metrics. These values are also referenced in the main text to support interpretation of the results in the Results section. In addition, we have specified appropriate units for abundance (individuals per core) to ensure clarity and comparability across studies.

Regarding the comparability of Control and Short treatments, we have addressed this comment in a previous reply. In brief, all plots were sampled at the same time after the 7-day experimental period. The Control plots remained under ambient conditions without any imposed stressor, making it very unlikely that macrobenthic fauna changed substantially during the additional two days. Previous studies in similar estuarine habitats indicate that short-term natural variability over two days does not significantly alter macrobenthic community composition or functional traits compared to experimental heating effects.

Line 321-323: Repetition of results line 238.

**Reply:** Thank you for comment. In this revised version of the manuscript, we have reviewed the text and rephrased the statement to avoid duplication. In addition, we have expanded the example and general idea presented to enhance the discussion without repeating previously reported findings. Lines 390-397.

Line 323-325: Where is the data showing the elevated densities of scavengers? Please also elaborate on the cascading effect and emerging of organisms and suggest more references on this discussion. Especially, as it was stated earlier (line 313) that burrowing into the sediment is a behavioural adaptation in response to heat stress. The suggested references, discussing stranded carrion and carrion cycling, are only vaguely applicable here.

**Reply:** Thank you for highlighting this point. In the revised manuscript, we have added the data values supporting the densities of scavengers and also included a complementary example about filter/suspension feeders. The data values are also now presented in the supplementary materials (S2-S6 Tables), which include quantitative Community Weighted Mean (CWM) trait data for all trait modalities, including scavengers and filter/suspension feeders. We have also referenced these values in the Discussion section to support interpretation. Lines 394-397

Additionally, we have expanded the discussion to elaborate on the cascading effects and the emergence of organisms under heat stress. Specifically, we explain how short-term heatwaves can alter resource availability and behavioral responses, such as increased scavenging activity, which may influence trophic interactions and nutrient cycling. We have supplemented this section with additional references. Lines 398-403.

Line 330: Please clarify “… were less responsive…” compared to?

**Reply:** As suggested by the reviewer, this sentence was clarified. We also provide a more detailed explanation on the functional metric patterns observed. Lines 416-426.

Line 333-334: Be specific about which species. Furthermore, if the majority of the reduction in abundance is attributed to the three dominant species (as noted in line 316), it would be valuable to discuss their functional traits and roles in the community in order to place these changes into a broader context of community trait redundancy.

**Reply:** As suggested by the reviewer, in this revised version of the manuscript we have specified the relevant functional traits and macrobenthic taxa. Lines 422-426.

**References**

Gruner DS, Bracken ME, Berger SA, Eriksson BK, Gamfeldt L, et al. 2017. Effects of experimental warming on biodiversity depend on ecosystem type and local species composition. *Oikos* **126**(1): 8–17.

Patrício J, Neto JM, Teixeira H, Salas F, Marques JC. 2009. The robustness of ecological indicators to detect long-term changes in the macrobenthos of estuarine systems. *Marine Environmental Research* **68**(1): 25–36.

Ritter C, Montagna PA, Applebaum S. 2005. Short-term succession dynamics of macrobenthos in a salinity-stressed estuary. *Journal of Experimental Marine Biology and Ecology* **323**(1): 57–69.

Ysebaert T, Herman PM. 2002. Spatial and temporal variation in benthic macrofauna and relationships with environmental variables in an estuarine, intertidal soft-sediment environment. *Marine Ecology Progress Series* **244**: 105–124.
