# Supplementary material for: Redundancy of macrobenthic functional traits boosts resilience to a simulated heatwave
Source: PLoS One. 2026 Jan 12;21(1):e0340819. doi: 10.1371/journal.pone.0340819 (PMC12795362; doi:10.1371/journal.pone.0340819)
Supplement: Attachment — Submitted filename: Reply_point_by_point_PlosOne_re_revision_OL_DL.docx [file pone.0340819.s013.docx]

**Ref.: Ms. No. PONE-D-25-44021R1**

Redundancy of macrobenthic functional traits boosts resilience to a simulated heatwave

Dear Academic Editor Marcos Rubal García,

We thank you and the reviewers for constructive comments and the opportunity to revise our manuscript. In this new revision, we have addressed all comments and suggestions from both reviewers.

**RESPONSE TO THE REVIEWER’S COMMENTS**

**REVIEWER #1:**

I would like to thank the authors for carefully revising this manuscript, which I still believe may be an excellent contribution to the study and application of functional metrics, necessary to understand and predict how ecosystems may respond to the effects of global climate change.

**Reply:** Thank you for your positive assessment of our manuscript and for recognizing its potential contribution to advancing the study and application of functional metrics in predicting ecosystem responses to climate change.

Overall, the authors have thoroughly revised the manuscript by checking and correcting inconsistencies between the main text and the graphs. They have also properly integrated the manuscript with the required information and explanations regarding the experimental design and methodologies used, as requested. Furthermore, they have provided sound and valid counterarguments to the comments expressed in the introduction and discussion sections, and have rightly added supplementary information, allowing for a better elaboration of the data obtained and a clearer interpretation of the results.

**Reply:** Thank you for your positive evaluation of the revisions. Your feedback reinforces the importance of the review process, and we are pleased that the revised manuscript now offers a clearer interpretation of the results and a more robust presentation of the study.

In light of the comments and revisions made, I support the acceptance of the manuscript. However, I ask the authors to further check and clarify a few aspects that have emerged after a careful assessment of the data provided in the supplementary materials, which were not previously presented. I consider this further minor revision a crucial step towards ensuring the scientific validity and consistency of the study, and ultimately necessary for acceptance.

**Reply:** Thank you for your supportive feedback. We appreciate your careful assessment of the supplementary materials and agree that addressing these remaining points will improve the manuscript. In the revised version, we have thoroughly checked the supplementary data and clarified all aspects highlighted in your comments.

Materials and Methods

Data analysis

Line 188 and S1 Table: Firstly, thank you for providing this table. The authors mentioned that no data standardisation or normalisation was performed. Clarification of the values presented in the tables is required. Is this combined matrix obtained by merging species abundances with functional categories? Such merging would provide more reliable quantitative information regarding the functional aspects of the community. Otherwise, this table appears to provide only qualitative information related to the presence of each species or taxon and their relative affinity for certain trait modalities, without weighting by the number of individuals (i.e. abundance) found. Consequently, the values are likely to be low in dispersion, and there may be no need for standardisation or normalisation. In my opinion, a quantitative trait analysis would be more informative in the context of this study, and I would suggest considering this type of analysis, as it could add robustness to the work. I would like to clarify that the procedure performed by the authors is correct, but it may be less informative and contextualised, since variations in species abundances are primarily indicative of changes in community structure when exposed to disturbance. If the authors wish to consider my suggestion to obtain a new merged species-trait matrix weighted by abundance, it is necessary to ensure consistency in the calculation of other functional metrics, including CWM. In this case, standardisation or normalisation may also be required. For example, the high abundance of the amphipod Paracorophium excavatum may not be comparable with the lower abundances of other species (e.g. Paradoneis lyra).

**Reply:** Thank you for raising this point. The values presented in Table S1 are only the fuzzy coding applied to each taxon. Yet, for all our statistical analyses we used the suggested quantitative data obtained from combining the macrobenthic abundances with their associated functional traits. This approach ensures that the analysis incorporates quantitative information on the functional aspects of the community rather than relying solely on presence/absence data. This step was performed using the FD package in R software, which performs the combining step prior to providing the outcomes used in our analysis. This information is presented in lines 199-200. In the revised manuscript, we have added new text to further clarify the process, lines 200-202.

Thank you also for your suggestions regarding the standardization/normalization of the data. While we appreciate the consideration of standardization or normalization, we determined that these steps were not necessary for our dataset. The combined macrobenthic–trait matrix weighted by abundance did not exhibit overdispersion (shade plots), and applying standardization would have artificially reduced the true variability among treatments, potentially masking ecologically meaningful differences. Our approach preserves the natural variation in macrobenthic abundances and trait expression, which is critical for accurately interpreting functional responses to heat stress.

Lines 205-209: It is stated that “Euclidean distance” was used for both structural metrics (i.e. abundances, S, and H’) and functional metrics (FRic, FEve, FDis, FR, and CWM). However, as the functional trait analysis includes both categorical traits (e.g. feeding mode, movement method, etc.) and continuous traits (e.g. body size), the literature recommends using the Gower dissimilarity index (Gower, 1971; Teichert et al., 2017). Euclidean distance is typically applied only to continuous (environmental) variables.

**Reply:** Thank you for your comment. We acknowledge the recommendation to use Gower dissimilarity when combining categorical and continuous traits. However, in our analysis, we used Euclidean distance because the data input for PERMANOVA consisted of single-variable metrics (e.g., FRic, FEve, FDis, FR, CWM) derived from the combined species–trait matrix, rather than raw trait data mixing categorical and continuous variables. This approach aligns with the protocols suggested by Anderson (2001) for PERMANOVA, where Euclidean distance is commonly applied to univariate or single-metric datasets in ecological studies. Using Euclidean distance ensures consistency with these established practices and is appropriate for the type of data analyzed in this study.

Results

Lines 299-301: The FRic values are surprisingly high given the number of species found (25). A high FRic indicates a very wide trait space, and this index is usually closely linked to species richness.

**Reply:** Thank you for highlighting this point. We have crosschecked the values and calculations and are correct. The high FRic values observed are due to the way Functional Richness is calculated: FRic considers not only taxa richness but also the distribution of taxa abundances within the multidimensional trait space. Because our analysis uses abundance-weighted trait data, taxa with higher abundances contribute more strongly to the expansion of the trait space, resulting in higher FRic values even with a moderate number of taxa.

We did identify the wrong citation in text of a supplementary table “S5”, we have changed to appropriate supplementary table “S6”, line 302.

Lines 304-305: Please verify that the FEve values exactly match those provided in Table S6. (FEve values range from 0 to 1.)

**Reply:** Thank you for pointing this out. You are correct that the FEve values in the text did not match those in Table S6. In this revised version of the manuscript, we have carefully reviewed and corrected the values to ensure consistency with the supplementary table, lines 307-308.

**REVIEWER #2:**

The manuscript has experienced a thorough revision after its initial submission and the authors have addressed all remarks and I particularly appreciate the effort invested into the data provision and strengthening the ecological context of the study. Most of the issues have been sufficiently resolved, but a few comments remain. The referred line numbers below are according to the track change document.

**Reply:** Thank you for your positive assessment of the revisions and for acknowledging the effort invested in improving the manuscript. We are pleased that the changes have strengthened the ecological context and addressed most of the previous concerns. In this revised version, we have addressed each of the suggestions.

Lines 61: Please clarify these contrasting statements. Line 61: “Estuarine macrobenthic organisms are likely well adapted to cope with extreme weather events such as MHWs …” and line 69: “… these organisms are predicted to be more sensitive to temperature extremes”

**Reply:** Thank you for highlighting this point. We have revised the text to clarify the apparent contradiction. In the previous revision we added text explaining that “while intertidal macrobenthic communities are generally adapted to natural fluctuations such as tidal cycles and seasonal changes, they remain highly sensitive to extreme and prolonged stressors beyond their usual environmental range, such as marine heatwaves”. In this revised version of the manuscript, we have reviewed the text and provided further clarification: “Estuarine macrobenthic organisms have the potential to cope with extreme weather events such as MHWs for short periods [30-32], however, negative impacts may occur when organisms face prolonged periods of elevated temperature that exceed their tolerance levels [31, 33-35].” and “These organisms are predicted to be more sensitive to temperature as global warming continues to increase, particularly when the temperature extremes are prolonged [36-39].”. Lines 56-57 and 64-65.

Lines 165 (Macrobenthic fauna sampling): Please add the sampling dates for the macrofauna. The current description is confusing due to inconsistencies between the manuscript and the revision responses. Specifically, the manuscript describes experimental treatment days, while the responses alternatively state that (i) Control and Long (7-day exposure) were sampled after 7 days, with the Short (5-day exposure) treatment sampled after 5 days, and (ii) that all treatments were sampled simultaneously after the 7-day experimental period. Please clarify.

**Reply:** Thank you for highlighting this concern. As suggested by the reviewer, in this revised version of the manuscript we have added the specific sampling dates for the macrofauna to remove any ambiguity, lines 160-161. To clarify, the Control and Long treatments (7-day exposure) were sampled on Day 7, while the Short treatment (5-day exposure) was sampled on Day 5.

Lines 242–249 and Fig. 2: Please clarify whether temperature differences between the Short and Long treatments were statistically tested. In addition, it is difficult to identify from Fig. 2 when the OTC chambers were deployed during the Short treatment, as temperatures appear consistently higher than the Control across all days. If the Short treatment was sampled at the same time as the other treatments (23/02/2024?) – Would you have an explanation why the temperature was elevated on 17 and 18/02/2024?

**Reply:** Thank you for this comment. Yes, temperature differences between the Control, Short and Long treatments were statistically tested. The details of these analyses are provided in the Methods section (lines 176–182), and the relevant PERMANOVA results are presented in the Results section (lines 236-237). Additionally, we confirm that the data shown in Figure 2 (and in general the data analyzed) correspond only to the periods when the OTC chambers were deployed. Furthermore, as stated in the previous comment, short treatment was not sampled at the same time as the other treatments.

Lines 394–403: While the example of functional traits (e.g. suspension feeders, predators, scavengers) in the Results section (lines 287–291) is appropriate, caution is needed when interpreting and linking these traits, as the discussion establishes an apparent link between them that is not directly supported by the data. To my knowledge, a large body of literature indicates that a common response of benthic invertebrates to excessive heat is deeper burrowing into the sediment (agreeing with your suggestion in line 377), rather than surfacing, unless driven by hypoxia, which I don’t suppose to be the case here. The manuscript suggests surfacing of animals and links this to a trophic cascade of increased resource availability for scavengers feeding on carrion and nutrient cycling. Given the lack of direct observations or data on animal movement in this study, such an interpretation is not sufficiently supported and should be reconsidered or more cautiously framed. In addition, given the availability of data now, I ask the authors to re-examine Table S5. The reported trait proportions (3% in Control, 5% in Short, and 6.8% in Long) appear to correspond to predators rather than scavengers (0.4%, 0.8%, 0.8%, respectively). Please verify this point and revise the text and discussion accordingly.

**Reply:** Thank you for highlighting this important concern. We agree that, given the lack of direct observations or behavioral data in our study, the interpretation regarding surfacing and its link to trophic cascades should be presented more cautiously. In the revised manuscript, we have reframed this section to clarify that these ideas are speculative and based on patterns reported in previous literature rather than direct evidence from our experiment. Specifically, we now state that while heat stress can influence resource availability and trophic interactions, the potential mechanisms such as surfacing or increased scavenging, remain hypothetical in this context and require further investigation. This adjustment ensures that our discussion acknowledges the limitations of the data while still providing ecological context supported by relevant references. Lines 393-396.

We also thank you for drawing our attention to re-examine Table S5. After carefully re-examining Table S5, we identified an error: the trait modality omnivore was inadvertently omitted, which caused all other feeding trait modality values to be shifted. We have corrected this mistake, and the updated Table S5 now includes the complete set of feeding trait modalities. The revised values align with those described in the text and discussion.

Line 413: According to Mouillot et al. (2013, “A functional approach reveals community responses to disturbances”), FDis is generally expected to decrease under disturbance due to the loss of functions. In contrast, in this study, FDis significantly increases. The authors may wish to contextualize these results – Could the observed pattern be related to the openness of the study system?

**Reply:** Thank you for this insightful comment. We agree that the observed increase in FDis under disturbance contrasts with the general expectation outlined by Mouillot et al. (2013). In Mouillot et al. (2013) paper is explained that many disturbances select against particular trait values and often reduce functional dispersion (FDis) through loss of functional type. However, FDis can increase in systems that are open to immigration (allowing colonisation by species with novel trait combinations), where disturbance intensity, duration, or spatial scale promote coexistence among complementary trait types, or when opportunistic/invasive species with novel traits enter the community. This pattern has been reported in other open systems where functional redundancy and trait variability buffer against disturbance effects. In this revised version of the manuscript, we have added text to contextualize our unexpected increase in FDis and point to possible mechanisms (connectivity/openness, colonisation/invasion, and disturbance regime). We used relevant references to support our statements, lines 409-420.

Recommended (constructive, clearly optional)

Given that the authors have conducted extensive analyses in related work, resulting in two previously published articles, it would be valuable to further interpret the observed changes in functional traits in relation to the metabolic responses of Austrovenus stutchburyi and/or, potentially, to changes in greenhouse gas fluxes. For example, the significantly increased proportion of bioirrigators in the Long treatment (and the near-significant increase in the Short treatment) could be discussed in the context of the elevated CO₂ and CH₄ influxes reported in Douglas et al. (2025). This additional interpretation is optional and, although speculative, I find it highly interesting. It could further enhance the ecological integration of the study.

**Reply:** Thank you for this thoughtful suggestion. We agree that linking functional trait changes to metabolic responses and greenhouse gas fluxes would be highly interesting and could enhance ecological integration. However, we believe that we do not currently have the right data to support these arguments, and as you noted, such interpretation would be speculative. Additionally, this level of integration is beyond the scope of the present study.

That said, we appreciate the idea and consider it an important direction for future research. We are planning a “version 2.0” of this experiment in February 2026, focused on an *Austrovenus stutchburyi* dominated sandflat community, where we aim to combine functional trait analyses with metabolic measurements and greenhouse gas flux assessments. This integrated approach will allow us to generate robust data to address these questions in a more comprehensive manner.
